# Rottlerin: Structure Modifications and KCNQ1/KCNE1 Ion Channel Activity

**DOI:** 10.1002/cmdc.202000083

**Published:** 2020-05-05

**Authors:** Marco Lübke, Julian A. Schreiber, Thang Le Quoc, Florian Körber, Jasmin Müller, Sivatharushan Sivanathan, Veronika Matschke, Janina Schubert, Nathalie Strutz‐Seebohm, Guiscard Seebohm, Jürgen Scherkenbeck

**Affiliations:** ^1^ Faculty of Mathematics and Natural Sciences University Wuppertal 42119 Wuppertal Germany; ^2^ Institut für Genetik von Herzerkrankungen University Hospital Münster Albert-Schweitzer-Campus 1 48149 Münster Germany; ^3^ Department of Chemistry Hue University 34 Le Loi St. Hue City Vietnam; ^4^ Medical Faculty, Institute of Anatomy Department of Cytology Ruhr University Bochum 44801 Bochum Germany

**Keywords:** mode of action, natural product rottlerin, potassium channel KCNQ1 activator, total synthesis

## Abstract

The slow delayed rectifier potassium current (I_Ks_) is formed by the KCNQ1 (K_v_7.1) channel, an ion channel of four α‐subunits that modulates KCNE1 β‐subunits. I_Ks_ is central to the repolarization of the cardiac action potential. Loss of function mutation reducing ventricular cardiac I_Ks_ cause the long‐QT syndrome (LQTS), a disorder that predisposes patients to arrhythmia and sudden death. Current therapy for LQTS is inadequate. Rottlerin, a natural product of the kamala tree, activates I_Ks_ and has the potential to provide a new strategy for rational drug therapy. In this study, we show that simple modifications such as penta‐acetylation or penta‐methylation of rottlerin blunts activation activity. Total synthesis was used to prepare side‐chain‐modified derivatives that slowed down KCNQ1/KCNE1 channel deactivation to different degrees. A binding hypothesis of rottlerin is provided that opens the way to improved I_Ks_ activators as novel therapeutics for the treatment of LQTS.

## Graphical abstract

**The polyphenolic natural product** rottlerin, isolated from Kamala powder, has been used for centuries in traditional Indian medicine. Among other biological effects, rottlerin has been shown to activate KCNQ1 potassium channels. Rottlerin analogues were synthesized and evaluated electrophysiologically in *X. laevis* oocytes. A molecular modeling based model was developed to explain the binding‐mode of rottlerin and other KCNQ1 ligands.

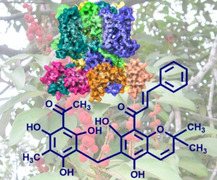

## Introduction

**Figure 1:**
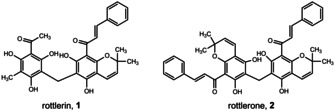
Structures of rottlerin (1) and rottlerone (2).

The kamala tree, *Mallotus philippensis* (Lam.) Muell. Arg. var. philippensis (Euphorbiaceae) is widely distributed in Southeast Asia. The granular hairs on the surface of the fruits of *M. philippensis* are covered with a reddish powder, which has been used since ancient times in traditional Indian medicine and as a natural red dye.^1^, ^2^ Numerous chemical constituents have been isolated from kamala powder, the majority of which belongs to the family of polyphenols.^3^ A diverse range of biological activities has been reported for kamala powder and extracts thereof, including anthelmintic, antibacterial, antiplasmodial, and cytotoxic eﬀects.^4^, ^5^, ^6^, ^7^ Among various dimeric phloroglucinol compounds, isolated from *M. philippensis*, only the major constituent rottlerin (1), also known as mallotoxin, has been studied more intensively for its biological potential (Figure 1).

Inhibitory activities have been reported for several kinases such as p38‐regulated/activated protein kinase (PRAK), mitogen‐activated protein kinase‐activated protein kinase 2 (MAP‐KAPK‐2), protein kinase B (PKB, Akt), Ca^2+^/calmodulin‐dependent protein kinase (CaMK), and protein kinase C‐delta (PKC‐δ).^8^, ^9^ In 2005 Zakharov and coworkers first described an agonistic activity on a BK potassium channel (SLO‐1).^10^ Recently, we identified rottlerin as a potent KCNQ1 and KCNQ4 activator.^11^ There is a well‐recognized need for improved therapies in K_V_7 based channelopathies.^12^ A drug that activates the respective KCNQ1 K^+^ channel, might be useful for treatment of inherited forms of potentially lethal LQTS, high blood pressure, diabetes and hearing impairment.^12^, ^13^ Currently, there is no selective, highly potent published KCNQ1/KCNE1 activator known, which is available for the clinical use of LQTS. In this paper, we describe the first rottlerin derivatives, obtained by derivatization of the natural product and total synthesis. KCNQ1/KCNE1 activities as well as first structure activity relationships are studied and discussed.

## Results and Discussion

### Syntheses of rottlerin derivatives

Though, the structure of rottlerin is known for decades, the first two total syntheses were published only recently. Both syntheses rely on a convergent, two building‐block approach, consisting of a chromene and a phloroglucinol unit which are coupled in the final steps via a methylene bridge. In the Wang^14^ synthesis, three different protecting groups are used, which renders this route less straightforward compared to the Kumar^15^ route. Since rottlerin is known to be unstable under intermediate acidic and basic conditions, the choice of the protective group strategy and the method for the introduction of the methylene bridge between the chromene and phloroglucinol building blocks are essential for success.^16^, ^17^

**Scheme 1:**
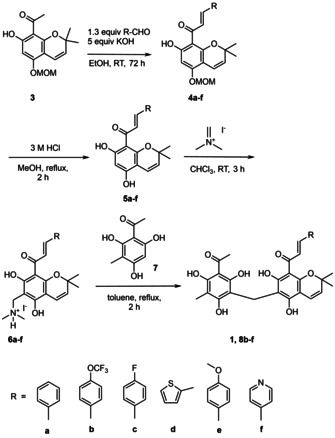
Total synthesis of rottlerin (**1**) and derivatives **8 b**–**f**.

After manifold efforts to develop a more efficient rottlerin synthesis, we finally embarked with minor modifications on the Kumar synthesis for the preparation of rottlerin derivatives **5 b**–**d** (Scheme 1). Due to our experience, only the MOM‐group was compatible with all steps of the synthesis, even if the yields (**5 a**–**d**) of the deprotection reaction were not optimal (Table 1). The acidic conditions (3 M HCl) led to dimerization and decomposition of the chromene units, especially in case of the electron‐rich methoxy substituent (**5 d**). With more diluted HCl the deprotection became inacceptable slow, again with concomitant formation of side products. Remarkably, even the structurally related MEM‐protective group turned out to be unsuitable. With TFA or HCl (6 M) either no reaction or decomposition of the chromene unit was observed. Cleavage with BBr_3_ led to the unexpected formation of rottlerone (**2**, Figure 1) by intermediate formation of an oxonium species, which attacks a second chromene molecule. Both, methyl ether or silyl ether protective groups provided problems with selective cleavage and/or stability in different stages of the synthesis. Anyway, the construction of chromene building blocks **5 a**–**f** and phloroglucinol **7** (Scheme 1) is standard chemistry in polyphenol and flavonoid synthesis.^18^, ^19^, ^20^, ^21^, ^22^

**Table 1:** Yields for chromene derivatives and final coupling steps.

	Yield [%]				
	4	5	6	8*	1*
a	73	32	79		23
b	89	27	98	25	
c	62	17	83	17	
d	58	36	53	19	
e	31	–			
f	–

* low yields due to losses during chromatographic purification.

Though, many polyphenolic natural products with a methylene bridge are known and have been synthesized, the introduction of an appropriate one‐carbon linker has developed into a considerable challenge for the synthesis of rottlerin. In our hands, all formaldehyde based standard procedures for coupling symmetric polyphenol building blocks under basic or acidic conditions failed for the unsymmetrical rottlerin.^23^, ^24^ We generally observed either decomposition or the preferred formation of symmetrical products, regardless of whether the methylol group was pre‐formed on the chromene or phloroglucinol moiety or a mixture of building blocks was used. Trials to introduce a chloromethyl or bromomethyl group into methyl ether‐protected acetyl phloroglucinol and subsequent palladium catalyzed cross‐coupling reactions to introduce the chromene unit were unsuccessful, too. Already the formation of halogenomethyl derivatives failed due to an unexpected replacement of the acetyl group by a second chloromethyl or bromomethyl group. Also, the use of MOMCl as a one‐carbon source under acidic conditions was not successful.^25^ Only Eschenmoser's salt, used by Kumar, gave satisfactory yields in the coupling reaction of the phloroglucinol and the chromene moieties.^23^, ^26^ Noticeably, the dimethylaminomethyl group has to be introduced into the chromene unit. The corresponding phloroglucinol derivative is accessible only in low yields and does not couple to the chromene unit.

**Figure 2:**
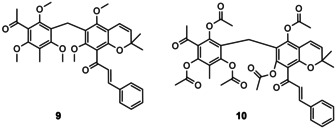
Rottlerin derivatives by modification of the natural product.

Finally, we were able to synthesize rottlerin in multi‐milligram amounts and the first derivatives (**8 b**–**d**) with modified side chain arenes. However, rottlerin can be obtained more straightforward in gram quantities by extraction from natural kamala powder, following a literature procedure.^27^ Reaction of the natural product with dimethylsulfate yielded pentamethyl rottlerin (**9**) in 73 % yield. Reaction with acetic anhydride gave penta‐acetyl rottlerin (**10**) in 92 % yield, respectively (Figure 2).

#### Compounds 8 b–d increase steady‐state activation of KCNQ1/KCNE1 channels

**Figure 3:**
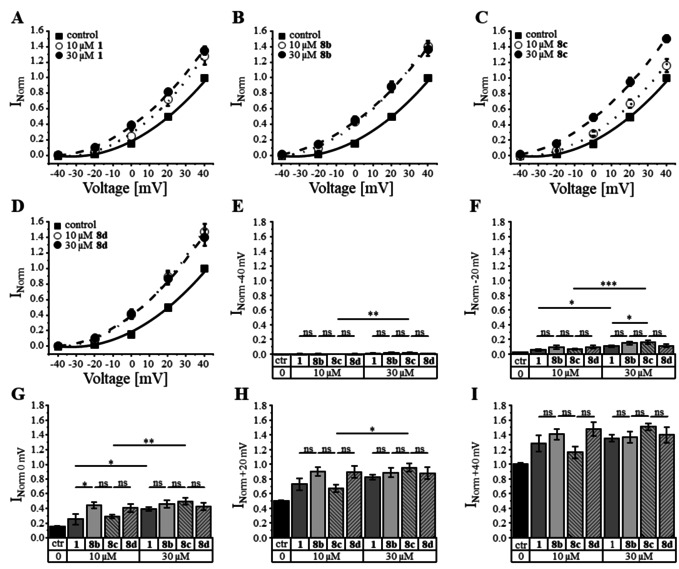
A)–D) Voltage dependent activation of KCNQ1/KCNE1 channels in absence (control, ctr) and presence of rottlerin (**1**) and analogs **8 b**–**d**. Compounds were applied at 10 and 30 μM, and the effect on steady‐state activated current amplitude was assessed at different voltages. Current amplitudes were normalized to the mean of amplitudes at +40 mV in absence of test compounds (control) E)‐I) Mean of normalized current amplitudes±SEM of different voltage steps in absence (ctr) and presence of rottlerin (**1**) and **8 b**–**d**. The number of independent experimental data points are given in Table 2. Significance of mean differences was determined by one‐way ANOVA and post‐hoc mean comparison Tukey test (ns: *p*>0.05, *p<0.05, **p<0.01, ***p<0.001).

Compounds **8 b**–**d** were tested at heteromeric KCNQ1/KCNE1 channels, expressed in *Xenopus laevis* oocytes. Channel sensitivity to tested compounds was determined by using the two‐electrode voltage clamp (TEVC) technique. KCNQ1/KCNE1 channels were activated by sequentially applied pulse protocols. Compounds **8 b**–**d** were tested at concentrations of 0.1–30 μM (see the Experimental Section). Only concentrations of more than 10 μM **8 b**–**d** could significantly increase activating currents (Table 2). Therefore, only 10 μM and 30 μM of compounds **8 b**–**d** were compared with the activity of rottlerin (**1**) to study the influence of newly incorporated substituents (Figure 3A–D). All three derivatives and rottlerin are nearly equally effective (Figure 3E–I). Only at lower test pulses of −20 mV and 0 mV, **8 b** and **8 c** increased activation current significantly more than rottlerin (Figure 3F–G). Interestingly, only compound **8 c** shows a significant dose‐dependent rise of activation current in the range of −40 mV to +20 mV for the tested concentration levels suggesting a right‐shift of the dose–response curve (Figure 3E–3I).

**Table 2:** normalized currents of KCNQ1/KCNE1 expressing oocytes in absence and presence of compounds **8 b**–**d** or rottlerin (**1**).

Conc.	Comp.	Voltage				
[μM]	(n)	−40 mV	−20 mV	0 mV	20 mV	40 mV
ctr	−(127)	0.00±0.00; –	0.03±0.00; –	0.16±0.01; –	0.50±0.01; –	1.00±0.02; –
0.1	**8 b** (5)	0.00±0.00; ns	0.03±0.01; ns	0.20±0.04; ns	0.56±0.07; ns	1.02±0.10; ns
	**8 c** (5)	0.00±0.00; ns	0.04±0.01; ns	0.20±0.04; ns	0.54±0.07; ns	1.00±0.10; ns
	**8 d** (4)	0.00±0.00; ns	0.05±0.01; ns	0.24±0.02; ns	0.65±0.04; ns	1.17±0.05; ns
0.3	**8 b** (5)	0.00±0.00; ns	0.03±0.01; ns	0.19±0.04; ns	0.53±0.07; ns	0.98±0.10; ns
	**8 c** (5)	0.00±0.00; ns	0.03±0.01; ns	0.15±0.04; ns	0.49±0.05; ns	1.04±0.05; ns
	**8 d** (3)	0.00±0.00; ns	0.03±0.01; ns	0.18±0.04; ns	0.55±0.06; ns	1.06±0.07; ns
1.0	**8 b** (5)	0.00±0.00; ns	0.04±0.01; ns	0.22±0.04; ns	0.58±0.07; ns	1.05±0.09; ns
	**8 c** (5)	0.00±0.00; ns	0.03±0.00; ns	0.19±0.02; ns	0.59±0.06; ns	1.16±0.12; ns
	**8 d** (5)	0.00±0.00; ns	0.03±0.01; ns	0.19±0.02; ns	0.55±0.04; ns	1.04±0.07; ns
3.0	**8 b** (5)	‐0.01±0.01; ns	0.03±0.02; ns	0.18±0.05; ns	0.49±0.10; ns	0.92±0.13; ns
	**8 c** (5)	‐0.01±0.00; ns	0.03±0.01; ns	0.18±0.02; ns	0.55±0.04; ns	1.06±0.06; ns
	**8 d** (5)	0.00±0.00; ns	0.03±0.01; ns	0.18±0.04; ns	0.56±0.07; ns	1.08±0.11; ns
10.0	**1** (5)	0.00±0.00; ns	0.06±0.01; *	0.26±0.00; *	0.73±0.08; **	1.28±0.11; **
	**8 b** (5)	0.01±0.00; ns	0.10±0.03; ***	0.44±0.04; ***	0.90±0.06; ***	1.41±0.07; ***
	**8 c** (5)	0.00±0.00; ns	0.07±0.01; ***	0.29±0.03; ***	0.67±0.05; ns	1.16±0.08; ns
	**8 d** (4)	0.00±0.01; ns	0.10±0.02; ***	0.41±0.05; ***	0.89±0.08; ***	1.48±0.09; ***
30.0	**1** (18)	0.01±0.00; ***	0.11±0.01; ***	0.39±0.03; ***	0.82±0.04; ***	1.35±0.05; ***
	**8 b** (5)	0.02±0.01; ***	0.15±0.02; ***	0.46±0.05; ***	0.89±0.07; ***	1.37±0.08; ***
	**8 c** (5)	0.02±0.01; ***	0.16±0.03; ***	0.50±0.04; ***	0.96±0.05; ***	1.51±0.05; ***
	**8 d** (4)	0.01±0.00; ns	0.11±0.02; ***	0.42±0.05; ***	0.88±0.08; ***	1.40±0.11; ***

Values are given as mean of 3–127 independent oocytes±SEM. Number of independent oocytes (n) is given for each compound and concentration. Significance of mean differences compared to absence of compound (ctr) was determined by one‐way ANOVA and post hoc mean comparison Tukey test (p>0.05 ns; p<0.05*; p<0.01**; p<0.001***).

#### Compounds 8 b–d deactivate KCNQ1/KCNE1 channels

**Figure 4:**
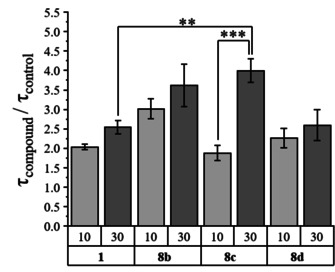
Relation of KCNQ1/KCNE1 deactivation time constants in the presence of rottlerin (**1**) and compounds **8 b**–**8 d** (*τ*_compound_) compared to absence of compounds (*τ*_control_). Time constants of deactivation were determined as described in the Experimental Section. Values are given as mean ±SEM. The significance of mean differences was determined by one‐way ANOVA and post‐hoc mean comparison Tukey test (ns: *p*>0.05, *p<0.05, **p<0.01, ***p<0.001).

KCNQ1 channels coexpressed with the KCNE1 subunit deactivate slowly in a voltage dependent manner and deactivation was reported to be slowed by native rottlerin.^11^, ^28^ Therefore, we tested how rottlerin analogs **8 b**–**d** impacted deactivation of KCNQ1/KCNE1 channels. Time dependent decay, indicative of channel deactivation was analyzed by single‐exponential fitting in absence and presence of compounds **8 b**–**d** and compared to deactivation in presence of rottlerin (**1**; see the Experimental Section). At concentrations of 10 μM, the ratio of deactivation constants of analogs **8 b**–**d** shows no significant differences compared to **1**, indicating similar efficiency at KCNQ1/KCNE1 channels (Figure 4). However, at concentrations of 30 μM time constant ratio of compound **8 c** was significantly higher than the ratio for 30 μM rottlerin (**1**) indicating an increased slowdown of channel deactivation.

**Figure 5:**
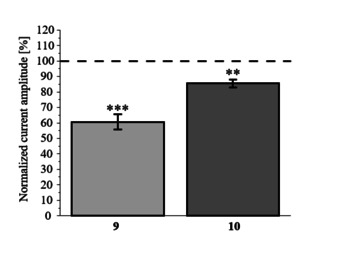
Normalized current amplitudes of KCNQ1/KCNE1‐expressing oocytes in the presence of 30 μM of compounds **9** and **10**. The effect of derivatives on KCNQ1/KCNE1 currents was determined as described above and are shown as mean±SEM (*n*=9–12, unpaired t‐test **p<0.01, ***p<0.001).

To evaluate the influence of pentamethylation and penta‐acetylation, compounds **9** and **10** were also tested at KCNQ1/KCNE1 expressing oocytes. Interestingly, rottlerin derivatives **9** and **10** decreased the normalized current amplitudes suggesting an antagonistic activity instead of agonism (Figure 5). However, these compounds are not suited as LQT‐drug candidates since antagonistic activity at KCNQ1/KCNE1 would amplify the pathology of LQT syndrome.

### Molecular modeling studies

A cryo‐EM structure of a KCNQ1/calmodulin (CaM) closed pore complex of *Xenopus laevis* was published by MacKinnon in 2017 (PDB ID: 5VMS).^28^ This construct shared 78 % sequence homology with the human KCNQ1 channel and was used by DeSilva to develop a binding‐model for rottlerin by *in silico* binding‐site prediction.^29^ These simulations of a single KCNQ1 subunit suggested, that rottlerin binds to the S4/S4–S5 region, stabilizing the channel by hydrogen bond formation to Arg243.

This hypothesis is well supported by the fact that mutation of Arg243 to alanine made KCNQ1 completely insensitive to rottlerin.^29^ In a seminal paper, MacKinnon provided the structural basis of human KCNQ1 modulation and gating.^30^ In particular, the exact binding‐site of PIP_2_ was elucidated in a KCNQ1‐KCNE3‐CaM complex. Essentially, this complex functions as a PIP_2_ ligand‐gated ion channel, which on binding of PIP_2_ undergoes large conformational changes and pore‐opening.

In our own, foregoing work based on a homology model of the open tetrameric channel, we suggested that rottlerin binds to the same or an overlapping binding site around the amino acid Ile337 as the benzodiazepinone R−L3 (**14**). A flexible *in silico* docking of rottlerin gave the highest docking scores for a crevice, located between VSD and PD (S5‐S6 outer face). This region overlaps with the R−L3 activator **14** binding‐site. Mutation of Ile337 in the center of the R−L3 binding site to valine, rendered the KCNQ1 channel insensitive to rottlerin.

**Figure 6:**
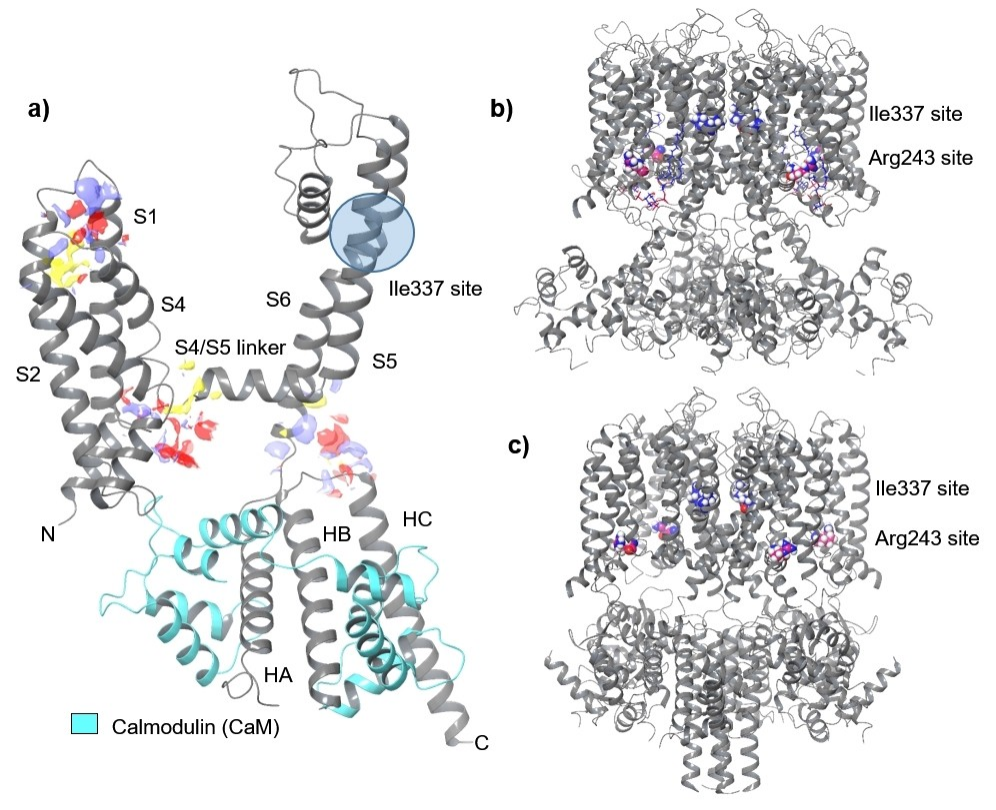
a) Structure of a KCNQ1 subunit and potential binding sites for small molecules. b) open KCNQ1 channel (6V01, PIP_2_ bound, human), c) closed KCNQ1 channel (6UZZ, human).

We first performed an unbiased virtual binding site mapping (Schrödinger modeling package 11.5) on a single subunit of the closed channel structure (*Xenopus laevis*, PDB ID: 5VMS), which has also been used by DeSilva. The mapping revealed ten potential binding sites for small molecules, seven of which however were located at the interfaces between the channel subunit and CaM. The three remaining binding sites were identified in the S1/S4 domain, between the S4 helix and S4S/S5 linker, including Arg243, and in the S6/HC region (Figure 6). Remarkably, no binding‐site was identified around Ile337. Similar results were obtained with the single units of the closed (PDB ID: 6UZZ) and opened (PDB ID: 6V01) channel structure of hK_v_7.1.

**Figure 7:**
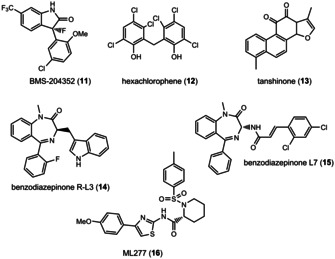
Structures of published KCNQ1 ligands.

Our KCNQ1 *in silico* ligand library consisted of rottlerin (**1**), derivatives **8 b**–**d**, and known ligands such as the agonistic benzodiazepinone R−L3 (**14**), the antagonistic benzodiazepinone L7 (**15**), the natural product tanshinone (**13**), the thiazole ML277 (**16**) and others (Figure 7). All ligands were prepared with the ligprep routine of the Schrödinger modeling suite, to generate different tautomers and protonation states between pH 5–9. All docking experiments were performed with Glide in extra precision mode.

Dockings into the three putative binding sites were performed with a single KCNQ1 subunit obtained from the PDB structure 5VMS (Table 3). The Arg243 site turned out to be specific for rottlerin and derivatives **8 b**–**d**, demonstrated by the significantly higher docking scores compared to all non‐rottlerin compounds. The preferred poses of rottlerin and derivatives **8 b**–**d** form tight clusters, which underlines the relevance of the Arg243 binding site and thus confirms the results of DeSilva.^28^ The S6/HA/HB site shows only minor affinity and in particular, the S1/S4 site appears not relevant for rottlerin binding at all. Cluster formation of rottlerin and derivatives **8 b**–**d** poses in the S1/S4 and the S6/HA/HB site is less pronounced which again favors the Arg243 site of the KCNQ1 subunit.

**Table 3:** Docking scores of ligand library for single KCNQ1 subunit derived from PDB structure 5VMS.

Compound	Arg243 site	S1/S4 site	S6/HA/HB site
rottlerin (**1**)	−6.6	−1.8	−4.5
thiophene rottlerin (**8 d**)	−6.4	−2.3	−4.9
CF_3_Ophenyl rottlerin (**8 b**)	−6.2	−2.4	−5.1
fluorophenyl rottlerin (**8 c**)	−6.1	−6.2	−3.4
BMS204352 (**11**)^31^	−3.5	−2.6	−2.2
hexachlorophene (**12**) ^[32]^	−3.2	−4.5	−2.3
pentamethoxy rottlerin (**9**)	−2.9	−1.9	−0.7
tanshinone (**13**)^33^	−2.8	−3.4	−1.9
penta‐acetoxy rottlerin (**10**)	−2.3	n.f.	n.f.
benzodiazepinine R−L3 (**14**)^34^	−1.6	−2.2	−2.2
benzodiazepinone L7 (**15**)^35^	−1.6	−2.1	−1.5
ML277 (**16**)^36^	−1.4	−3.0	−2.9

Numbers represent the calculated docking‐scores. Docking scores are in kcal/mol: the difference in binding for two compounds is ΔΔ*G*=Δ*G*2–Δ*G*1=*RT*×ln (*K*_D2_ /*K*_D1_); at 25 °C, a difference in binding score of 1.36 kcal/mol corresponds to a tenfold difference in *K*_D_; more negative values indicate a higher predicted affinity. Docking scores in the range of 4 or below are usually not significant. n.f.: not found.

The recently published structure of a human KCNQ1‐KCNE3‐CaM‐PIP_2_ (PDB ID: 6V01) complex allows a more thorough insight into the binding mode of rottlerin as an activator of this specific potassium channel.^30^ Important to note, the PIP_2_ bound structure 6V01 has an open pore while the KCNQ1 structures 5VMS and 6UZZ represent the potassium channels in the closed state. Our modeling studies were performed with the complete tetrameric channel, which is relevant in particular for 6V01, since the binding pocket of PIP_2_ is formed by two neighboring subunits.

**Figure 8:**
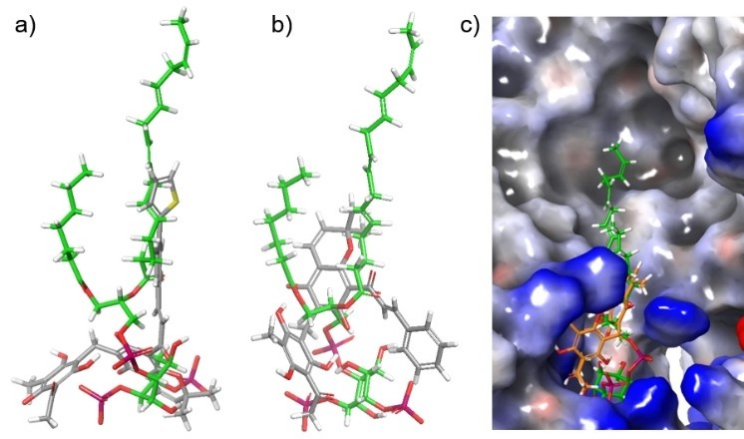
a) Superposition of thiophene rottlerin **8 d** and PIP_2_. b) Superposition of rottlerin and PIP_2_. c) Orientation of rottlerin (orange) and PIP_2_ (green) in the Arg243 site. According to the cryo‐EM structure 6V01, PIP_2_ contains heptanoic acid in sn1 position and (*5E*,*8E*,*11E*,*14Z*)‐hexadecatetraenoic acid in position sn2.

In general, the tendencies resemble the docking studies with 5VMS. The Arg243 site of both channel structures shows a considerably higher affinity for rottlerin and its phenyl‐substituted derivatives than for other known KCNQ1 ligands. Again, pentamethoxy‐rottlerin **9** and penta‐acetoxy rottlerin **10** had either no or only a modest affinity *in silico*. Interestingly, rottlerin and derivatives **8 b**–**d**, express a slightly higher affinity to the closed KCNQ1 potassium channel (Table 4). All poses collected for rottlerin form a single cluster, which is oriented to the inositol headgroup of PIP_2_. The chromene unit of rottlerin serves as a kind of pincer, which directs the styrene side chain into a cavity occupied by the inositol‐4‐phosphate group (Figure 8). The electron‐rich trihydroxyphenyl side chain of rottlerin is placed into a cationic binding‐pocket in direct neighborhood of the inositol residue with one phenolic hydroxy group oriented to the inositol 3‐hydroxy group. The chromene unit itself imitates the branching of the two PIP_2_ alkyl side chains.

**Table 4:** Docking scores of ligand library for Arg243 and Ile337 sites performed with PDB structures 6UZZ and 6V01 (complete channels).

Compound	6UZZ Arg243	6V01 Arg243	6UZZ Ile337	6V01 Ile337
thiophene rottlerin (**8 d**)	−9.6	−8.8	−5.2	−8.7
rottlerin (**1**)	−9.0	−8.2	−4.5	−6.3
CF_3_Ophenyl rottlerin (**8 b**)	−9.0	−8.3	−5.1	−9.4
fluorophenyl rottlerin (**8 c**)	−8.9	−7.5	−4.3	−6.4
tanshinone (**13**)	−5.6	−4.5	−6.7	−5.8
hexachlorophene (**12**)	−5.5	−4.9	−5.9	−4.6
benzodiazepinone L7 (**15**)	−4.9	−5.5	−8.0	−5.8
BMS204352 (**11**)	−4.6	−3.6	−6.3	−7.0
ML277 (**16**)	−4.4	−4.3	−6.4	−6.9
benzodiazepinone R−L3 (**14**)	−3.8	−3.8	−6.0	−6.6
pentamethoxy rottlerin (**9**)	−1.7	−1.8	−4.8	n.f.
penta‐acetoxy rottlerin (**10**)	n.f.	−3.5	−3.8	n.f.

Rottlerin is fixed in the Arg243 site by hydrogen bonds to Arg192, Lys196 and a cation‐π interaction between the styrene phenyl ring and Lys196. This binding mode offers an obvious explanation for the low affinities of pentamethoxy rottlerin **9** and the penta‐acetoxy derivative **10**. Both rottlerin derivatives lost an important hydrogen bond and additionally in derivative **10**, the electronic situation of the phenolic oxygens has been altered by acetylation. Remarkably, derivatives **8 b** and **8 d** orient differently in the binding pocket compared to rottlerin. Since the styrene pocket is too small to accommodate a substituted benzene ring, the preferred orientation of both compounds is now an optimal match of the more lipophilic arene side chains with the unsaturated side chain of PIP_2_. In this arrangement, the chromene unit serves as a mimic for the inositol‐ring with the pyrane oxygen situated close to or in the pocket of the inositol‐4‐phosphate group, while the partially negatively charged trihydroxyphenyl ring imitates the 5‐phosphate residue.

**Figure 9:**
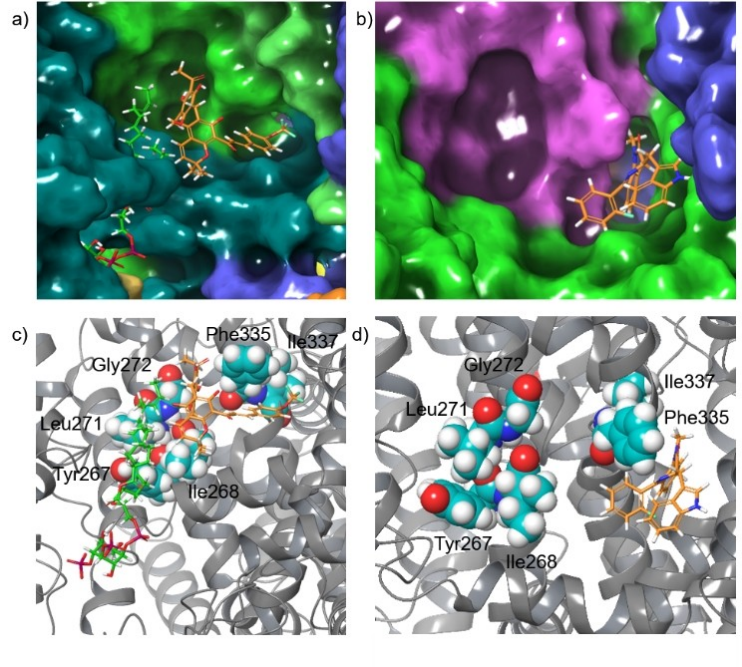
a) Open‐channel binding‐pocket around Ile337 (PDB ID: 6V01) with bound trifluoromethoxyphenyl rottlerin **8 b**. Colors code protein chains. b) Closed‐channel Ile337 binding pocket (PDB ID: 6UZZ) with bound benzodiazepinone **14**. Colors code protein chains. c) Open‐channel structure with residues found important for rottlerin and benzodiazepinone **14** binding (PDB ID: 6V01). d) Closed‐channel structure with residues found essential for rottlerin and benzodiazepinone **14** binding (PDB ID: 6UZZ).

Contrary to the single subunits, the tetrameric KCNQ1 channels from PDB structures 6UZZ and 6V01 form well‐defined binding pockets around Ile337 by contribution of two neighboring subunits in the open and the closed state. The pocket in the open state (PDB ID: 6V01) overlaps with the binding site of the PIP_2_ unsaturated chain and has a high affinity for rottlerin and derivatives **8 b**–**d**. It consists of two interconnected crevices, which accommodate the two aromatic side chains of rottlerin (Figure 9). In the closed state (PDB ID: 6UZZ), the two sites are smaller and more separated by Phe335, which protrudes into the channel, connecting the two sites. This reduces the affinity for rottlerin and derivatives **8 b**–**d** by a factor of 5–10. Instead, the binding of more compact molecules such as the benzodiazepinones or the natural product tanshinone (**13**) is favored. The significance of the open‐channel pocket is also underlined by pentamethoxy and penta‐acetyl rottlerin **9** and **10**, both of which are not found as binders. As we have already demonstrated, mutation of Ile337 to a sterically more demanding Val changes the shape of the binding‐pocket in a way, that neither the styrene phenyl ring of rottlerin nor the benzodiazepinone **14** can be accommodated anymore.

Based on these findings, it is reasonable to assume, that rottlerin mimics in a way PIP_2_. Rottlerin binds to the open and/or closed KCNQ1 potassium channel, which results in channel opening or keeping the channel open. Rottlerin and even more the derivatives **8 b** and **8 d** show a considerable affinity and distinct selectivity for the Ile337 site of the open channel. Presumably, binding to that site exerts a similar effect as the agonistic benzodiazepinone **14**, which stabilizes the open state of the KCNQ1 channel. Overall, modifications of the phenyl residue of the styrene side chain appear to be of limited benefit for more powerful rottlerin derivatives, since the binding‐pocket for rottlerin can accommodate only an unsubstituted benzene ring. Larger phenyl substituents cause a reorientation of the rottlerin derivative in the Arg243 binding site, now mimicking the unsaturated PIP_2_ side chain, which contributes only via weak lipophilic interactions to the overall binding energy.

## Conclusion

The functional effect, the increment of deactivation time and activation current for compounds **8 b** and **8 d** showed similar activity at 10 and 30 μM compared to rottlerin (**1**), while compound **8 c** showed slightly, but not significant reduced activity at 10 μM and slightly increased activity at 30 μM. This might indicate a right‐shifted dose‐response curve for **8 c** and a preference for aromatic systems with high electron density at this position. However, at high concentration of 30 μM compound **8 c** was more potent in slowing down KCNQ1/KCNE1 channel deactivation.

In the context of therapy of the long QT syndrome, activation of KCNQ1/KCNE1 channels was discussed as a therapy option. Due to the lack of drugs on the market that activate the native KCNQ1/KCNE1 channel, novel compounds would be desired. Rottlerin is such an activator of I_Ks_ 11, 28 However, further scaffold optimization is needed to gain higher selectivity, improved kinetic profile and agonistic activity at KCNQ1/KCNE1 channels. Synthetic rottlerin and its derivatives are all active as KCNQ1/KCNE1 channel activators. Interestingly, derivatives **8 b**–**8 d** preserve channel activating efficiency, but show kinetic alterations, suggesting that future chemical modifications may provide fine‐tuned pharmacological effects.

## Experimental Section

**Isolation of rottlerin (1)**: Rottlerin was isolated from *Mallotus phillippinensis* Kamala powder, purchased from Kremer Pigmente GmbH, Germany according to a literature procedure.^27^ Kamala powder (15 g) was refluxed in boiling benzene for 7 h. After filtration of the insoluble material rottlerin was obtained from the solution by precipitation. Recrystallization yielded rottlerin (680 mg) of the same purity as a commercial reference sample.

### Syntheses

**Abbreviations**: Cyh, cyclohexane; EDDA, ethylenediamine diacetate; MOM, methoxymethyl; sat., saturated; TMS, tetramethylsilane

**General**: IR spectra were recorded on a Bruker ALPHA FTIR spectrometer. ^1^H and ^13^C NMR spectra were recorded on Bruker Avance III 600 and Bruker Avance 400 spectrometers operating at 600 and 400 MHz (^1^H) respectively and 150 and 100 MHz (^13^C) respectively. Accurate mass determinations were achieved with a Bruker micrOTOF mass spectrometer. The reactions were monitored by TLC carried out on Macherey Nagel silica gel plates (60F‐254) or Merk silica gel 60 RP‐18 F254 plates using UV light and aqueous solution of KMnO_4_, K_2_CO_3_, NaOH and heat as the visualizing agents. HPLC analysis was conducted on a PerfectSil Target ODS‐3 HD 5 μm 100×4.6 mm column using an Agilent 1100 instrument. Reagents and solvents were purchased from commercial sources and used without further purification, unless otherwise stated. CH_2_Cl_2_ was dried with a MB‐SPS‐800 solvent purification system. MeOH was redistilled from magnesium turnings. Reactions were stirred magnetically under an argon atmosphere unless otherwise stated.

**General procedure for preparation of chalcones 4**: To a solution of 1‐[7‐Hydroxy‐5‐(methoxymethoxy)‐2,2‐dimethyl‐chromen‐8‐yl]ethenone (**3**) (1 equiv) in ethanol (15 mL) were added potassium hydroxide (5 equiv) and the aryl aldehyde (1.3 equiv). The reaction mixture was stirred for 3 days at room temperature. At completion, a saturated NH_4_Cl‐solution was added and the mixture war partitioned between water (50 mL) and ethyl acetate (50 mL). The aqueous layer was extracted with ethyl acetate (2×30 mL). The combined organic extracts were washed with brine (15 mL), dried over Na_2_SO_4_, and concentrated under reduced pressure. The crude products were purified by flash chromatography over silica gel to provide the desired compound (**4**).

**(*E*)‐1‐[7‐Hydroxy‐5‐(methoxymethoxy)‐2,2‐dimethyl‐chromen‐8‐yl]‐3‐phenyl‐prop‐2‐en‐1‐on (4 a)**: Compound **4 a** was obtained from 1‐[7‐hydroxy‐5‐(methoxymethoxy)‐2,2‐dimethyl‐chromen‐8‐yl]ethenone (**3**) and benzaldehyde. 288 mg, yield: 73 %. ^1^H NMR (400 MHz, CDCl_3_): δ (ppm)=13.98 (s, 1H), 8.13 (d, *J*=15.6 Hz, 1H), 7.80 (d, *J*=15.6 Hz, 1H), 7.65–7.62 (m, 2H), 7.47–7.38 (m, 3H), 6.65 (d, *J*=10.0 Hz, 1H), 6.28 (s, 1H), 5.50 (d, *J*=10.0 Hz, 1H), 5.25 (s, 2H), 3.52 (s, 3H), 1.58 (s, 6 H). ^13^C NMR (100 MHz, CDCl_3_): δ (ppm)=193.0, 166.9, 158.7, 155.9, 142.3, 135.6, 130.1, 129.0, 128.3, 127.5, 124.9, 116.8, 107.0, 103.6, 95.3, 94.3, 78.0, 56.5, 28.0. HPLC‐MS (ESI): m/z (%): 365.2 [M^−^−H] (100), 366.2 [M^−^] (18). HR‐MS (ESI): *m/z* found=365.1396, calcd for C_121_H_21_O_5_=365.1394. IR (cm^−1^): 3068, 2972, 2958, 2907, 2827, 1642, 1582, 1546. Rf: 0.52 (CH/EE 19 : 1).

**(*E*)‐1‐[7‐Hydroxy‐5‐(methoxymethoxy)‐2,2‐dimethyl‐chromen‐8‐yl]‐3‐[4‐(trifluoromethoxy) phenyl]prop‐2‐en‐1‐one (4 b)**: Product **4 b** was obtained from 1‐[7‐hydroxy‐5‐(methoxymethoxy)‐2,2‐dimethyl‐chromen‐8‐yl]ethenone (**3**) and 4‐(trifluoromethoxy)‐benzaldehyde. 293 mg, yield: 89 %. ^1^H NMR (400 MHz, CDCl_3_): δ (ppm)=13.89 (s, 1H), 8.08 (d, *J*=15.8 Hz, 1H), 7.75 (d, *J*=15.5 Hz, 1H), 7.64 (d, *J*=8.7 Hz, 2H), 7.28 (d, *J*=8.1 Hz, 2H), 6,64 (d, *J*=9.9 Hz, 1H), 6.28 (s, 1H), 5.51 (d, *J*=10.0 Hz), 5.25 (s, 2H), 3.52 (s, 3H), 1.57 (s, 6H). ^13^C NMR (100 MHz, CDCl_3_): δ (ppm)=192.6, 166.9, 158.9, 155.9, 150.2, 140.3, 134.2, 129.5, 128.3, 124.9, 121.2, 120.4 (q, *J*=258.7 Hz), 116.8, 106.9, 103.6, 95.3, 94.3, 78.1, 56.5, 28.1. HPLC‐MS (ESI): m/z (%): 449.2 [M^−^−H] (100), 450.2 [M^−^] (20). HR‐MS (ESI): *m/z* found=449.1219, calcd for C_23_H_20_F_3_O_6_=449.1217. IR (cm^−1^): 3115, 3079, 2974, 2958, 2915, 2836, 1637, 1587, 1545. Rf: 0.36 (CH_2_Cl_2_/CH 1 : 1).

**(*E*)‐3‐(4‐Fluorophenyl)‐1‐[7‐hydroxy‐5‐(methoxymethoxy)‐2,2‐dimethyl‐chromen‐8‐yl]prop‐2‐en‐1‐one (4 c)**: Product **4 c** was obtained from 1‐[7‐hydroxy‐5‐(methoxymethoxy)‐2,2‐dimethyl‐chromen‐8‐yl]ethenone (**3**) and 4‐fluorobenzaldehyde. 194 mg. yield: 62 %. ^1^H NMR (400 MHz, CDCl_3_): δ (ppm)=13.95 (s, 1H), 8.04 (d, *J*=15.7 Hz, 1H), 7.75 (d, *J*=15.7 Hz, 1H), 7.59–7.63 (m, 2H), 7.10–7.16 (m, 2H), 6.34 (d, *J*=10.0, 1H), 6.28 (s, 1H), 5.50 (d, *J*=10.0 Hz, 1H), 5.25 (s, 2H), 3.52 (s, 3 H), 1.57 (s, 6H). ^13^C NMR (100 MHz, CDCl_3_): δ (ppm)=192.8, 166.8, 163.4 (d, *J*=251.3 Hz), 158.7, 155.8, 141.0, 131.8 (d, *J*=3.4 Hz), 130.0 (d, *J*=8.5 Hz), 127.2, 124.8, 116.8, 116.1 116.1 (d, *J*=21.9 Hz), 106.9, 103.6, 95.3, 94.3, 78.1, 56.5, 28.0. HPLC‐MS (ESI): m/z (%): 385.1 [M^−^−H] (100), 386.1 [M^−^] (19). HR‐MS (ESI): *m/z* found=407.1264, calcd for C_22_H_21_F_1_Na_1_O_4_=407.1265 IR (cm^−1^): 2974, 2958, 2906, 1634, 1582, 1544, 1507. Rf: 0.24 (CH/EE 19 : 1).

**(*E*)‐1‐[7‐Hydroxy‐5‐(methoxymethoxy)‐2,2‐dimethyl‐chromen‐8‐yl]‐3‐(2‐thienyl)prop‐2‐en‐1‐one (4 d)**: Product **4 d** was obtained from 1‐[7‐hydroxy‐5‐(methoxymethoxy)‐2,2‐dimethyl‐chromen‐8‐yl]ethanon (**3**) and thiophen‐2‐carbaldehyde. 148 mg, 58 % yield. ^1^H NMR (400 MHz, CDCl_3_): δ (ppm)=14.05 (s, 1H), 8.00 (d, *J*=15.3 Hz), 7.94 (d, *J*=7.94 Hz), 7.40 (d, *J*=4.9 Hz, 1H), 7.32 (d, *J*=3.44 Hz), 7.10 (dd, *J*=5.1 Hz, *J*=3.6 Hz, 1H), 6.63 (d, *J*=10.4 Hz), 6.26 (s, 1H), 5.50 (d, *J*=10.4 Hz), 5.24 (s, 2H), 3.51 (s, 3H), 1.61 (s, 6H). ^13^C NMR (100 MHz, CDCl_3_): δ (ppm)=192.4, 166.9, 158.6, 155.8, 141.4, 135.1, 131.7, 128.3, 128.2, 126.5, 124.5, 116.6, 106.8, 103.6, 95.2, 94.3, 78.1, 56.5, 27.9. HPLC‐MS (ESI): m/z (%): 371.2 [M^−^−H] (100), 372.2 [M^−^] (17). HR‐MS (ESI): *m/z* found=395.0926, calcd for C_20_H_20_Na_1_O_5_S_1_=395.0924. IR (cm^−1^): 3326, 3032, 2957, 1709, 1585, 1542. Rf: 0.47 (CH/EE 4 : 1).

**(*E*)‐1‐[7‐Hydroxy‐5‐(methoxymethoxy)‐2,2‐dimethyl‐chromen‐8‐yl]‐3‐(4‐methoxyphenyl)prop‐2‐en‐1‐one (4 e)**: Compound **4 e** was obtained from 1‐[7‐hydroxy‐5‐(methoxymethoxy)‐2,2‐dimethyl‐chromen‐8‐yl]ethenone (**3**) and 4‐methoxybenzaldehyde. 11 mg, 31 % yield. ^1^H NMR (400 MHz, CDCl_3_): δ (ppm)=14.10 (s, 1H), 8.03 (d, *J*=15.7 Hz, 1H), 7.79 (d, *J*=15.7 Hz, 1H), 7.58 (d, *J*=8.7 Hz, 2H), 6.96 (d, *J*=8.7 Hz, 2H), 6.64 (d, *J*=9.8 Hz, 1H), 6.27 (s, 1H), 5.50 (d, *J*=9.9 Hz, 1H), 5.24 (s, 2H), 3.88 (s, 3H), 3.51 (s, 3H), 1.58 (s, 6H). ^13^C NMR (100 MHz, CDCl_3_): δ (ppm)=192.9, 166.9, 161.4, 158.5, 155.8, 142.5, 130.0, 128.4, 125.1, 124.8, 116.8, 114.5, 107.0, 103.6, 95.3, 94.3, 77.9, 56.5, 55.4, 28.0. HPLC‐MS (ESI): m/z (%): 395.2 [M^−^−H] (100), 396.2 [M^−^] (26). HR‐MS: *m/z* found=395.1502, calcd for C_23_H_23_O_6_=395.1500. IR (cm^−1^): 3108, 3065, 2992, 2968, 2937, 1683, 1593, 1546, 1508. Rf: 0.38 (CH/EE 5 : 1).

**General procedure for the deprotection of the MOM‐group**: Chalcones **4** were dissolved in methanol (30 mL) and a 3 M HCl solution (3 mL) was added. The reaction mixture was refluxed for 2 h. The reaction was quenched with a saturated NaHCO_3_‐solution (15 mL). The reaction mixture was partitioned between water (50 mL) and ethyl acetate (50 mL). The aqueous layer was extracted with ethyl acetate (2×30 mL). The combined organic phases were washed with brine, dried over Na_2_SO_4_, and concentrated under reduced pressure. The crude products were purified by flash chromatography over silica gel to give the deprotected compounds **5**.

**(*E*)‐1‐(5,7‐Dihydroxy‐2,2‐dimethyl‐chromen‐8‐yl)‐3‐phenyl‐prop‐2‐en‐1‐one (5 a)**: Compound **5 a** was obtained from (*E*)‐1‐[7‐hydroxy‐5‐(methoxymethoxy)‐2,2‐dimethyl‐chromen‐8‐yl]‐3‐phenyl‐prop‐2‐en‐1‐one (**4 a**). 78 mg, yield: 32 %. ^1^H NMR (400 MHz, CDCl_3_): δ (ppm)=14.11 (s, 1H), 8.12 (d, *J*=15.6 Hz, 1H), 7.79 (d, *J*=15.6 Hz, 1H), 7.65–7.60 (m, 2H), 7.47–7.39 (m, 2H), 6.60 (d, *J*=9.5 Hz, 1H), 6.00 (s, 1H), 5.51 (d, *J*=9.5 Hz, 1H), 1.58 (s, 6H). ^13^C NMR (100 MHz, CDCl_3_): δ (ppm)=193.0, 166.5, 158.0, 156.7, 142.4, 135.6, 130.1, 129.0, 128.3, 127.4, 124.9, 116.4, 106.6, 102.4, 96.4, 78.2, 28.0. HPLC‐MS (ESI): m/z (%): 321.2 [M^−^−H] (100), 322.2 [M^−^] (20). HR‐MS (ESI): *m/z* found=321.1130, calcd for C_20_H_17_O_4_=321.1132. IR (cm^−1^): 3233, 2973, 1593, 1546. Rf: 0.27 (CH/EE 8 : 2).

**(*E*)‐1‐(5,7‐Dihydroxy‐2,2‐dimethyl‐chromen‐8‐yl)‐3‐[4‐(trifluoromethoxy)phenyl]prop‐2‐en‐1‐one (5 b)**: Product **5 b** was obtained from (*E*)‐1‐[7‐hydroxy‐5‐(methoxymethoxy)‐2,2‐dimethyl‐chromen‐8‐yl]‐3‐[4‐(trifluoromethoxy)phenyl]prop‐2‐en‐1‐one (**4 b**). 69 mg, yield: 27 %. ^1^H NMR (400 MHz, CDCl_3_): δ (ppm)=14.02 (s, 1H), 8.08 (d, *J*=15.7 Hz, 1H), 7.74 (d, *J*=15.7 Hz), 7.63 (d, *J*=8.6 Hz, 2H), 7.28 (d, *J*=8.1 Hz, 2H), 6.60 (d, *J*=9.9 Hz, 1H), 6.21 (bs, 1H), 6.00 (s, 1H), 5.51 (d, *J*=9.8 Hz), 1.57 (s, 6H). ^13^C NMR (100 MHz, CDCl_3_): δ (ppm)=192.6, 166.5, 158.3, 156.7, 150.2, 140.4, 134.2, 129.5, 128.3, 124.8, 121.2, 120.4 (q, *J*=250.2 Hz), 116.4, 106.6, 102.4, 96.4, 78.3, 28.1. HPLC‐MS (ESI): m/z (%): 405.2 [M^−^−H] (100), 406.2 [M^−^] (22). HR‐MS (ESI): *m/z* found=405.0953, calcd for C_21_H_16_F_3_O_5_=405.0955. IR (cm^−1^): 3231, 3129, 2971, 2926, 2854, 1628, 1586, 1505. Rf: 0.24 (CH/EE 6 : 1).

**(*E*)‐1‐(5,7‐Dihydroxy‐2,2‐dimethyl‐chromen‐8‐yl)‐3‐(4‐fluorophenyl)prop‐2‐en‐1‐one (5 c)**: Product **5 c** was obtained from (*E*)‐3‐(4‐fluorophenyl)‐1‐[7‐hydroxy‐5‐(methoxymethoxy)‐2,2‐dimethyl‐chromen‐8‐yl]prop‐2‐en‐1‐one (**4 c**). 14 mg, yield: 17 %. ^1^H NMR (400 MHz, CDCl_3_): δ (ppm)=14.11 (s, 1H), 8.03 (d, *J*=15.4, 1H), 7.74 (d, *J*=15.4, 1H), 7.75–7.72 (m, 2H), 7.15–7.10 (m, 2H), 6.60 (d, *J*=9.9 Hz, 1H), 6.01 (s, 1H), 5.50 (d, *J*=9.9 Hz, 1H), 1.57 (s, 6H). ^13^C NMR (100 MHz, CDCl_3_): δ (ppm)=192.8, 166.4, 163.8 (d, *J*=251.3 Hz), 158.4, 156.7, 141.1, 131.8 (d, *J*=3.6 Hz), 130.0 (d, *J*=8.5 Hz), 127.2, 124.7, 116.5, 116.1 (d, *J*=21.9 Hz), 106.5, 102.5, 96.4, 78.2, 28.0. HPLC‐MS (ESI): m/z (%): 339.2 [M^−^−H] (100), 340.2 [M^−^] (19). HR‐MS (ESI): *m/z* found=339.1035, calcd for C_20_H_16_F_1_O_4_=339.1038. IR (cm^−1^): 3524, 3245, 2970, 2925, 1628, 1595, 1506. Rf: 0.17 (CH:EE 8 : 2).

**(*E*)‐1‐(5,7‐Dihydroxy‐2,2‐dimethyl‐chromen‐8‐yl)‐3‐(2‐thienyl)prop‐2‐en‐1‐one (5 d)**: Product **5 d** was obtained from (*E*)‐1‐[7‐hydroxy‐5‐(methoxymethoxy)‐2,2‐dimethyl‐chromen‐8‐yl]‐3‐(2‐thienyl)prop‐2‐en‐1‐one (**4 d**). 43 mg, yield: 36 %. ^1^H NMR (400 MHz, CDCl_3_): δ (ppm)=14.19 (s, 1H), 8.01 (d, *J*=15.3 Hz, 1H), 7.94 (d, *J*=15.4 Hz, 1H), 7.40 (d, *J*=5.3 Hz), 7.32 (d, *J*=3.5 Hz), 7.10 (dd, *J*=5.1 Hz, 3.6 Hz, 1H), 6.60 (d, *J*=9.9 Hz, 1H), 6.43 (bs, 1H), 6.00 (s, 1H), 5.51 (d, *J*=10.0 Hz, 1H), 1.62 (s, 6H,). ^13^C NMR (100 MHz, CDCl_3_): δ (ppm)=191.2, 166.6, 158.2, 156.6, 141.5, 135.2, 131.2, 128.3, 128.2, 126.5, 124.9, 116.4, 106.4, 102.4, 96.4, 78.3, 28.0. HPLC‐MS (ESI): m/z (%): 327.1 [M^−^−H] (100), 328.1 [M^−^] (18). HR‐MS (ESI): *m/z* found=326.0695, calcd for C_18_H_15_O_4_S_1_=326.0697. IR (cm^−1^): 3244, 3105, 3089, 2964, 2922, 1642, 1593, 1547, 1503. Rf: 0.30 (CH/EE 9 : 1).

**General procedure for reacting Eschenmoser's salt with chromene building blocks 5**:

To a solution of the deprotected chalcone (1 equiv) in chloroform (15 mL) was added Eschenmoser's salt (3 equiv). The reaction mixture was stirred for 3 h. The reaction mixture was then diluted with chloroform and a 1 M HCl solution (10 mL) was added. The aqueous phase was extracted with ethyl acetate (2×25 mL). The combined organic phases were washed with brine (10 mL), dried over Na_2_SO_4_, and concentrated under reduced pressure. The crude products **6** were used directly without further purification.

**[5,7‐Dihydroxy‐2,2‐dimethyl‐8‐[(*E*)‐3‐phenylprop‐2‐enoyl]chromen‐6‐yl]methyl‐dimethyl‐ammoniumiodide (6 a)**: Compound **6 a** was obtained from (*E*)‐1‐(5,7‐dihydroxy‐2,2‐dimethyl‐chromen‐8‐yl)‐3‐phenyl‐prop‐2‐en‐1‐one (**5 a**) and Eschenmoser's salt. 86 mg, yield: 79 %. HPLC‐MS (ESI): m/z (%): 380.2 [M^+^+H−HI] (34), 381.2 [M^+^−HI] (7), 335.1 [M^+^+H−HI−C_2_H_8_N] (100). HR‐MS (ESI): *m/z* found=380.1856, calcd for C_23_H_26_N_1_O_4_=380.1856.

**[5,7‐Dihydroxy‐2,2‐dimethyl‐8‐[*(E*)‐3‐[4‐(trifluoromethoxy)phenyl]prop‐2‐enoyl]chromen‐6‐yl]methyl‐dimethyl‐ammoniumiodide (6 b)**: Compound 6b was obtained from (*E*)‐1‐(5,7‐dihydroxy‐2,2‐dimethyl‐chromen‐8‐yl)‐3‐[4‐(trifluoromethoxy)phenyl]prop‐2‐en‐1‐one (5b) and Eschenmoser's salt. 64 mg, yield: 98 %. HPLC‐MS (ESI): m/z (%): 462.2 [M^−^−2H−HI] (100), 463.2[M^−^−H−HI] (23), 464.2 [M^−^−HI] (3). HR‐MS (ESI): *m/z* found=464.1677, calcd for C_24_H_25_F_3_N_1_O_5_=464.1679.

**[8‐[(*E*)‐3‐(4‐Fluorophenyl)prop‐2‐enoyl]‐5,7‐dihydroxy‐2,2‐dimethyl‐chromen‐6‐yl]methyl‐dimethyl‐ammoniumiodide (6 c)**: Compound **6 c** was obtained from (*E*)‐1‐(5,7‐dihydroxy‐2,2‐dimethyl‐chromen‐8‐yl)‐3‐(4‐fluorophenyl)prop‐2‐en‐1‐one (**5 c**) and Eschenmoser's salt. 32 mg, yield 83 %. HPLC‐MS (ESI): m/z (%): 339.2 [M^−^−H−HI−C_2_H_8_N] (100), 340.2 [M^−^−HI] (19). HR‐MS (ESI): *m/z* found=339.1035, calcd for C_20_H_16_F_1_O_4_=339.1038.

**[5,7‐Dihydroxy‐2,2‐dimethyl‐8‐[(*E*)‐3‐(2‐thienyl)prop‐2‐enoyl]chromen‐6‐yl]methyl‐dimethyl‐ammoniumiodide (6 d)**: Compound **6 d** was obtained from (*E*)‐1‐(5,7‐dihydroxy‐2,2‐dimethyl‐chromen‐8‐yl)‐3‐(2‐thienyl)prop‐2‐en‐1‐one (**5 d**) and Eschenmoser's salt. 36 mg, yield: 53 %. HPLC‐MS (ESI): m/z (%): 384.2 [M^−^−H−HI] (100), 385.2 [M^−^−HI] (26.6). HR‐MS (ESI): *m/z* found=386.1420, calcd for C_21_H_24_N_1_O_4_S_1_=386.1421.

**General procedure for couplings via Eschenmoser's salts 6**: A solution of the salt **6** (1 equiv) and 1‐(2,4,6‐trihydroxy‐3‐methyl‐phenyl)ethenone (1 equiv) in toluene was stirred for 1 h at 110 °C. At completion, the reaction mixture was concentrated under reduced pressure. The crude products were purified by flash chromatography over silica gel to obtain the final compound (**1**, **8**).

**(*E*)‐1‐[6‐[(3‐acetyl‐2,4,6‐trihydroxy‐5‐methyl‐phenyl)methyl]‐5,7‐dihydroxy‐2,2‐dimethyl‐chromen‐8‐yl]‐3‐phenyl‐prop‐2‐en‐1‐one, rottlerin (1)**: Rottlerin (1) was obtained from 1‐(2,4,6‐trihydroxy‐3‐methyl‐phenyl)ethenone (7) and [5,7‐dihydroxy‐2,2‐dimethyl‐8‐[(E)‐3‐phenylprop‐2‐enoyl]chromen‐6‐yl]methyl‐dimethyl‐ammoniumiodid (6a). 25 mg, yield: 29 %. ^1^H NMR (400 MHz, CDCl_3_): δ (ppm)=8.20 (d, 15.8 Hz, 1H), 7.85 (d, *J*=15.8 Hz, 1H), 7.65–7.60 (m, 2H), 7.47–7.39 (m, 3H), 6.68 (d, *J*=9.8 Hz, 1H), 5.50 (d, *J*=9,8 Hz), 3.82 (s, 2H), 2.73 (s, 3H), 2.10 (s, 3H), 1.56 (s, 6H). ^13^C NMR (100 MHz, CDCl_3_): δ (ppm)=204.1, 192.9, 162.8, 160.6, 159.6, 158.8, 156.6, 155.4, 143.2, 135.5, 130.3, 129.0, 128.4, 126.8, 125.1, 117.2, 106.5, 106.0, 105.3, 104.2, 103.7, 101.9, 78.2, 32.5, 28.0, 15.8, 7.5. HPLC‐MS (ESI): m/z (%): 515.3 [M^−^−H] (100), 516.3 [M^−^] (30). HR‐MS (ESI): *m/z* found=515.1713, calcd for C_30_H_28_O_8_=515.1711. IR (cm^−1^): 3234, 2954, 2922, 2851, 1710, 1603, 1555. Rf: 0.24 (CH/EE 2 : 1).

**(*E*)‐1‐[6‐[(3‐Acetyl‐2,4,6‐trihydroxy‐5‐methyl‐phenyl)methyl]‐5,7‐dihydroxy‐2,2‐dimethyl‐chromen‐8‐yl]‐3‐[4‐(trifluoromethoxy)‐phenyl]prop‐2‐en‐1‐one (8 b)**: Compound 8b was obtained from [5,7‐dihydroxy‐2,2‐dimethyl‐8‐[(*E*)‐3‐[4‐(trifluoromethoxy)phenyl]prop‐2‐enoyl]chromen‐6‐yl]methyl‐dimethyl‐ammoniumiodid (6b) and 1‐(2,4,6‐trihydroxy‐3‐methyl‐phenyl)ethenone (7). 6 mg, 25 % yield. ^1^H NMR (400 MHz, CDCl_3_): δ (ppm)=8.16 (d, *J*=15.5 Hz, 1H), 7.81 (d, *J*=15.7 Hz, 2H), 7.65 (m, 2H), 7.29 (m, 2H), 6.69 (d, *J*=9.9 Hz, 1H), 5.51 (d, *J*=9.9 Hz, 1 H), 3.84 (s, 2H), 2.74 (s, 3H), 2.11 (s, 3H), 1.55 (s, 6H). ^13^C NMR (100 MHz, CDCl_3_): δ (ppm)=204.1, 192.5, 162.8, 160.6, 159.6, 159.0, 155.4, 150.4, 141.3, 134.1, 129.7, 127.7, 125.1, 121.2, 120.4 (q, *J*=255.0 Hz) 117.2, 106.5, 105.9, 105.3, 104.2, 103.8, 101.9, 78.2, 32.5, 28.1, 15.8, 7.5. HPLC‐MS (ESI): m/z (%): 599.3 [M^−^−H] (100), 600.3 [M^−^] (30). HR‐MS (ESI): *m/z* found=599.1534, calcd for C_31_H_26_F_3_O_9_=599.134. IR (cm^−1^): 3246, 2961, 2925, 2855, 1630, 1598, 1507. Rf: 0.10 (CH/EE 2 : 1).

**(*E*)‐1‐[6‐[(3‐Acetyl‐2,4,6‐trihydroxy‐5‐methyl‐phenyl)methyl]‐5,7‐dihydroxy‐2,2‐dimethyl‐chromen‐8‐yl]‐3‐(4‐fluorophenyl)prop‐2‐en‐1‐one (8 c)**: Compound 8c was obtained from [8‐[*(E*)‐3‐(4‐fluorophenyl)prop‐2‐enoyl]‐5,7‐dihydroxy‐2,2‐dimethyl‐chromen‐6‐yl]methyl‐dimethylammonium‐iodide (6c) and 1‐(2,4,6‐trihydroxy‐3‐methyl‐phenyl)ethenone (7). 7 mg, yield: 21 %. ^1^H NMR (400 MHz, CDCl_3_): δ (ppm)=8.12 (d, *J*=15.4 Hz, 1H), 7.81 (d, *J*=15.5 Hz, 1H), 7.64–7.59 (m, 2H), 7.16–7.11 (m, 2H), 6.79 (d, *J*=9.9 Hz, 1H), 5.50 (d, *J*=9.9 Hz, 1H), 3.83 (s, 2H), 2.73 (s, 3H), 2.11 (s, 3H), 1.55 (s, 6H). ^13^C NMR (100 MHz, CDCl_3_): δ (ppm)=204.1, 192.7, 164.0 (d, *J*=251.3 Hz), 162.8, 160.6, 159.6, 158.8, 155.4, 142.0, 131.7 (d, *J*=3.2 Hz), 130.2 (d, *J*=8.7 Hz), 126.6, 125.0, 117.2, 116.2 (d, *J*=22.1 Hz), 106.5, 106.0, 105.2, 104.2, 103.8, 101.9, 78.2, 32.5, 28.0, 15.8, 7.4. HPLC‐MS (ESI): m/z (%): 533.3 [M^−^−H] (100), 534.3 [M^−^] (30). HR‐MS (ESI): *m/z* found=533,1615 calcd for C_30_H_26_F_1_O_8_=533.1617. IR (cm^−1^): 3247, 2954, 2923, 2852, 1599, 1507. 1507. Rf: 0.16 (CH/EE 2 : 1).

**(*E*)‐1‐[6‐[(3‐Acetyl‐2,4,6‐trihydroxy‐5‐methyl‐phenyl)methyl]‐5,7‐dihydroxy‐2,2‐dimethyl‐chromen‐8‐yl]‐3‐(2‐thienyl)prop‐2‐en‐1‐one (8 d)**: Compound 8d was obtained from [5,7‐dihydroxy‐2,2‐dimethyl‐8‐[(*E*)‐3‐(2‐thienyl)prop‐2‐enoyl]chromen‐6‐yl]methyl‐dimethyl‐ammoniumiodide (6d) and 1‐(2,4,6‐trihydroxy‐3‐methyl‐phenyl)ethenone (7). 13 mg, yield: 35 %. ^1^H NMR (400 MHz, CDCl_3_): δ (ppm)=8.07 (d, *J*=15.3 Hz, 1H), 7.98 (d, *J*=15.3 Hz, 1H), 7.43 (d, *J*=5.0 Hz, 1H), 7.34 (d, *J*=3.4 Hz, 1H), 7.10 (dd, *J*=5.3, 3.4, 1H), 6.67 (d, *J*=9.7 Hz, 1H), 5.50 (d, *J*=9.7 Hz, 1H), 3.82 (s, 2H), 2.73 (s, 3H), 2.10, (s, 3H), 1.58 (s, 6H). ^13^C NMR (100 MHz, CDCl_3_): δ (ppm)=204.1, 192.0, 162.9, 160.6, 159.6, 158.7, 155.3, 141.4, 136.1, 132.0, 128.7, 128.4, 125.8, 125.2, 117.1, 106.5, 106.0, 105.1, 104.3, 103.7, 101.9, 78.3, 32.5, 27.9, 15.8, 7.5. HPLC‐MS (ESI): m/z (%): 521.2 [M^−^−H] (100), 522.2 [M^−^] (30). HR‐MS (ESI): *m/z* found=521.1275, calcd for C_28_H_25_O_8_S_1_=521.1276. IR (cm^−1^): 3235, 2972, 2927, 1736, 1709, 1600, 1547. Rf: 0.27 (CH/EE 2 : 1).

### Molecular biology

**Materials and methods**: The KCNQ‐clones resembles the clone published by NCBI Annotation Project Accession numbers *XM_052604.2* (hKCNQ1) and NM_001127670.3 (hKCNE1). Molecular biological procedures were those described previously.^37^ In brief, cRNAs were generated by *in vitro* transcription with the Ambion T7 mMessage mMachine kit (Life Technologies. Darmstadt. Germany) from linearized cDNA templates.

**Two‐electrode voltage‐clamp (TEVC) in** ***Xenopus laevis*** **oocytes**: The standard TEVC procedures were similar as previously described. Defolliculated oocytes were obtained from EcoCyte Bioscience (Dortmund. Germany). Oocytes were injected with 8 ng KCNQ1‐WT and 4 ng KCNE1‐WT and stored for 3–4 days in Bath's solution containing (mmol L‐1): 88 NaCl, 1 KCl, 0.4 CaCl_2_, 0.33 Ca(NO3)_2_, 0.6 MgSO_4_, 5 TRIS−HCl, 2.4 NaHCO_3_ and supplemented with 80 mg L‐1 theophylline, 63 mg L‐1 benzylpenicillin, 40 mg L‐1 streptomycin and 100 mg L‐1 gentamycin. Standard TEVC recordings were performed at 22 °C with a Turbo Tec 10CX (NPI) amplifier combined with GePulse software for data acquisition. Macroscopic currents were recorded 3–4 days after injection in recording solution ND96 (NaCl 96 mM, KCl 2 mM, CaCl_2_ 1.8 mM, MgCl_2_ 1 mM, HEPES 5 mM, pH 7.4). Compound solutions were freshly prepared from 10 mM DMSO stock solutions. For maximal compatibility, all recording solutions including the control solution contained a final DMSO concentration of 0.3 %. Pipettes were filled with 3 M KCl and had resistances of 0.5–1.5 MΩ. Channel functions were analysed using the following pulse protocols: 
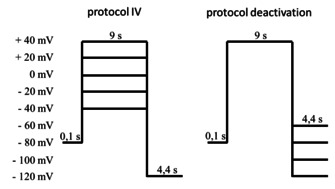


Pulse protocol IV was used to assess the voltage dependent activation of channel currents, while pulse protocol deactivation was used to assess the voltage dependent deactivation of channel currents. Both pulse protocols were sequential applied at the same oocyte to record reference currents (control). After completion, compound solutions were washed in and pulse protocols were applied again under presence of compound solution.

**Data analysis**: Electrophysiological data were recorded with GePulse and analyzed with accompanying software Ana (Dr. Michael Pusch. Genova. Italy). Data analysis was done using OriginPro 2018 (OriginLab Corporation. Northampton. MA. USA).

**Steady‐state activation**: Currents were normalized to the average current of the +40 mV pulses in absence of compounds (control) at the end of the 9 s for recordings of the same experiment (see representative current traces beneath). 
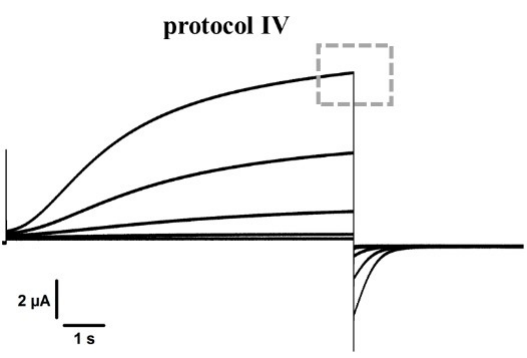


*I*_norm_ was calculated for every voltage step:

$$I_{norm}=\;\frac{I_{-\;40\;mV\;-\;+\;40\;mV}}{\bar{I_{+\;40\;mV}}}$$

The resulting normalized currents were fitted to following equitation:

$$y=\mathrm{ax}{}^{2}+\mathrm{bx}+c$$

### Deactivation


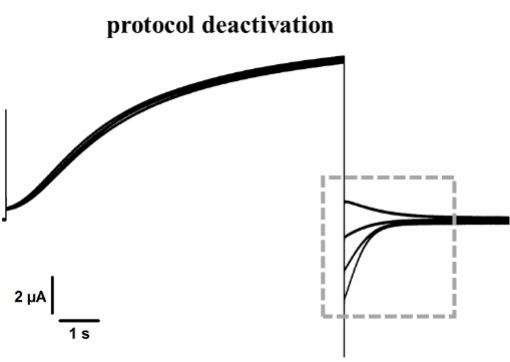
KCNQ1/KCNE1 channels deactivate due to hyperpolarization using pulse protocol tail current. This voltage dependent channel closure can be analyzed by means of exponential fits. For comparison of deactivation kinetics in presence of compounds, deactivation current trace was fitted to following equitation to determine time constant *τ*:

$$Y=A_{0}+A_{1}{}^{*}e^{\frac{-t}{\tau }}$$

Deactivation was fitted for current traces with voltage changes from +40 mV to −120 mV for all oocytes in absence (control) and presence of compounds. After determination of *τ*_D_ for all current traces, *τ*_compound_ was normalized to *τ*_control_:

$$\frac{\tau_{compound}}{\tau_{control}}$$

**Statistics**: Statistical significance of results was evaluated by one‐way ANOVA with post‐hoc mean comparison Tukey test or Student's t test and is indicated by asterisks (ns: *p*>0.05, *p<0.05, **p<0.01, ***p<0.001).

## Conflict of interest

The authors declare no conflict of interest.

## Supporting information

As a service to our authors and readers, this journal provides supporting information supplied by the authors. Such materials are peer reviewed and may be re‐organized for online delivery, but are not copy‐edited or typeset. Technical support issues arising from supporting information (other than missing files) should be addressed to the authors.

Supplementary

## References

[1] J. Sharma , R. Varma , Pharmacologyonline 2011, 3, 1256–1265.

[2] S. K. Jain , A. S. Pathania , S. Meena , R. Sharma , A. Sharma , B. Singh , B. D. Gupta , S. Bhushan , S. B. Bharate , R. A. Vishwakarma , J. Nat. Prod. 2013, 76, 1724–1730. 24041234 10.1021/np400433g

[3] M. Furusawa , Y. Ido , T. Tanaka , T. Ito , K. -I Nakaya , I. Ibrahim , M. Ohyama , M. Iinuma , Y. Takahashi , Helv. Chim. Acta 2005, 88, 1048–1058.

[4] I. P. Tripathi , P. Chaudhary , P. Pandey , World J. Pharm. Res. 2017, 6, 678–687.

[5] R. Tanaka , T. Nakata , C. Yamaguchi , S. Wada , T. Yamada , H. Tokuda , Planta Med. 2008, 74, 413–416. 18484534 10.1055/s-2008-1034347

[6] R. R. Kulkarni , S. G. Tupe , S. P. Gample , M. G. Chandgude , D. Sarkar , M. V. Deshpande , S. P. Joshi , Nat. Prod. Res. 2014, 28, 245–250. 24099509 10.1080/14786419.2013.843178

[7] V. J. Sharma , Plant Biochem. J. 2011, 20, 190–195.

[8] M. Gschwendt , H. J. Müller , K. Kielbassa , R. Zang , Biochem. Biophys. Res. Commun. 1994, 93–98. 10.1006/bbrc.1994.1199 8123051

[9] S. P. Soltoff , Trends Pharmacol. Sci. 2007, 28, 453–458. 17692392 10.1016/j.tips.2007.07.003

[10] S. I. Zakharov , J. P. Morrow , G. Liu , L. Yang , S. O. Marx , Biol. Chem. 2005, 280, 30882–30887. 10.1074/jbc.M505302200 15998639

[11] V. Matschke , I. Piccini , J. Schubert , E. Wrobel , F. Lang , J. Matschke , E. Amedonu , S. G. Meuth , T. Strünker , N. Strutz-Seebohm , B. Greber , J. Scherkenbeck , G. Seebohm , Cell. Physiol. Biochem. 2016, 40, 1549–1558. 27997884 10.1159/000453205

[12] Q. Xiong , Z. Gao , W. Wang , M. Li , Trends Pharmacol. Sci. 2008, 29, 99–107. 18206251 10.1016/j.tips.2007.11.010

[13] G. Seebohm , Mol. Pharmacol. 2005, 67, 585–588. 15608141 10.1124/mol.104.010173

[14] Y. Li , B. Yu , R. Wang , Tetrahedron Lett. 2016, 57, 1856–1859.

[15] K. K. C. Hong , G. E. Ball , D. StC Black , N. Kumar , J. Org. Chem. 2015, 80, 10668–10674. 26426936 10.1021/acs.joc.5b01827

[16] T. Backhouse , A. McGookin , J. Matchet , A. Robertson , E. Tittensor , J. Chem. Soc. 1948, 113–119. 18906365 10.1039/jr9480000113

[17] S. K. Chauthe , S. B. Bharate , S. Sabde , D. Mitra , K. K. Bhutani , I. P. Singh , Bioorg. Med. Chem. 2010, 18, 2029–2036. 20137956 10.1016/j.bmc.2010.01.023

[18] C. A. Elliger , Synth. Commun. 1985, 15, 1315–1324.

[19] K. Nakagawa-Goto , K.-H. Lee , Tetrahedron Lett. 2006, 47, 8263–8266.

[20] J. M. Keith , Tetrahedron Lett. 2004, 45, 2739–2742.

[21] M. J. Adler , S. W. Baldwin , Tetrahedron Lett. 2009, 50, 5075–5079.

[22] L. Xia , M. Narasimhulu , X. Li , J. J. Shim , Y. R. Lee , Bull. Korean Chem. Soc. 2010, 31, 664–669.

[23] A. Minassi , L. Cicione , A. Koeberle , J. Bauer , S. Laufer , O. Werz , G. Appendino , Eur. J. Org. Chem. 2012, 772–779.

[24] A. L. Eaton , S. Dalal , M. B. Cassera , S. Zhao , D. G. I. Kingston , J. Nat. Prod. 2016, 79, 1679–1683. 27228055 10.1021/acs.jnatprod.6b00347 PMC4924580

[25] T. D. Grayfer , P. Grellier , E. Mouray , R. H. Dodd , J. Dubois , K. Cariou , Org. Lett. 2016, 18, 708–711. 26828239 10.1021/acs.orglett.5b03676

[26] J. Schreiber , H. Maag , N. Hashimoto , A. Eschenmoser , Angew. Chem. Int. Ed. 1971, 10, 330–331;

[27] S. Dutt , J. Chem. Soc. Trans. 1925, 127, 2044–2052.

[28] J. Sun , R. MacKinnon , Cell 2017, 169, 1042–1050. 28575668 10.1016/j.cell.2017.05.019 PMC5562354

[29] A. M. De Silva , R. W. Manville , G. W. Abbott , Sci. Adv. 2018, 4, eaav0824. 30443601 10.1126/sciadv.aav0824 PMC6235520

[30] J. Sun , R. MacKinnon , Cell 2020, 180, 1–8. 31883792 10.1016/j.cell.2019.12.003 PMC7083075

[31] S. Jensen , CNS Drug Rev. 2002, 4, 353–360. 10.1111/j.1527-3458.2002.tb00233.x PMC6741660 12481191

[32] Y. Zheng , X. Zhu , P. Zhou , X. Lan , H. Xu , M. Li , Z. Gao , PLoS One 2012, 7, e51820, 1–9. 23251633 10.1371/journal.pone.0051820 PMC3520906

[33] D.-D. Sun , H.-C. Wang , X.-B. Wang , Y. Luo , Z.-X. Jin , Z.-C. Li , G.-R. Li , M.-Q. Dong , Eur. J. Pharmacol. 2008, 590, 317–321. 18573250 10.1016/j.ejphar.2008.06.005

[34] G. Seebohm , M. Pusch , J. Chen , M. C. Sanguinetti , Circ. Res. 2003, 93, 941–947. 14576198 10.1161/01.RES.0000102866.67863.2B

[35] G. Seebohm , J. Chen , N. Strutz , C. Culberson , C. Lerche , M. C. Sanguinetti , Mol. Pharmacol. 2003, 64, 70–77. 12815162 10.1124/mol.64.1.70

[36] M. E. Mattmann , H. Yu , Z. Lin , K. Xu , X. Huang , S. L. Meng , W. Owen , B. McManus , D. W. Engers , U. M. Le , M. Li , C. W. Lindsley , C. R. Hopkins , Bioorg. Med. Chem. Lett. 2012, 22, 5936–5941. 22910039 10.1016/j.bmcl.2012.07.060 PMC3433560

[37a] G. Seebohm , M. C. Sanguinetti , M. Pusch , J. Physiol. 2003, 552, 369–378; Erratum:14561821 10.1113/jphysiol.2003.046490 PMC2343369

[37b] G. Seebohm , M. C. Sanguinetti , M. Pusch , J. Physiol. 2004, 556, 1013.

