# Supplementary material for: Rottlerin: Structure Modifications and KCNQ1/KCNE1 Ion Channel Activity
Source: ChemMedChem. 2020 May 5;15(12):1078–88. doi: 10.1002/cmdc.202000083 (PMC7318133; doi:10.1002/cmdc.202000083)
Supplement: Supplementary file 1 — Supplementary [file CMDC-15-1078-s001.pdf]

ChemMedChem

Supporting Information

Rottlerin: Structure Modifications and KCNQ1/KCNE1 Ion Channel Activity

Marco Lübke[+], Julian A. Schreiber[+], Thang Le Quoc[+], Florian Körber, Jasmin Müller, Sivatharushan Sivanathan, Veronika Matschke, Janina Schubert, Nathalie Strutz-Seebohm, Guiscard Seebohm,* and Jürgen Scherkenbeck*© 2020 The Authors. Published by Wiley-VCH Verlag GmbH & Co. KGaA. This is an open access article under the terms of the Creative Commons Attribution License, which permits use, distribution and reproduction in any medium, provided the original work is properly cited.

Author Contributions

M.L. Methodology:Supporting; Writing - Original Draft:Supporting
J.S. Data curation:Supporting; Formal analysis:Supporting; Investigation:Supporting; Methodology:Supporting; Writing - Original Draft:Supporting
T.L. Methodology:Supporting
F.K. Methodology:Supporting
J.M. Methodology:Supporting
S.S. Methodology:Supporting
V.M. Methodology:Supporting
J.S. Methodology:Supporting
N.S.-S. Methodology:Supporting
G.S. Conceptualization:Equal; Data curation:Equal; Formal analysis:Equal; Methodology:Equal; Writing - Original Draft:Equal
J.S. Conceptualization:Equal; Methodology:Equal; Resources:Equal; Visualization:Equal; Writing - Original Draft:Equal; Writing - Review & Editing:Equal

SUPPLEMENTARY INFORMATION

Syntheses

Abbreviations
Cyh, cyclohexane; DCM, dichloromethane; EDDA, ethylenediamine diacetate; Et_2O, diethyl ether; EtOAc, ethyl acetate; MeOH, methanol; MOM, methoxymethyl; sat., saturated; TMS, tetramethylsilane.

General
IR spectra were recorded on a Bruker ALPHA FTIR spectrometer. ^{1}H- and ^{13}C-NMR spectra were recorded on Bruker Avance III 600 and Bruker Avance 400 spectrometers operating at 600 and 400 MHz (^{1}H) respectively and 150 and 100 MHz (^{13}C) respectively. Accurate mass determinations were achieved with a Bruker micrOTOF mass spectrometer. The reactions were monitored by TLC carried out on Macherey Nagel silica gel plates (60F-254) or Merk silica gel 60 RP-18 F254 plates using UV light and aqueous solution of $KMnO_4$, K_2CO_3, NaOH and heat as the visualizing agents. HPLC analysis was conducted on a PerfectSil Target ODS-3 HD 5 μm 100×4.6 mm column using an Agilent 1100 instrument. Reagents and solvents were purchased from commercial sources and used without further purification, unless otherwise stated. DCM was dried with a MB-SPS-800 solvent purification system. MeOH was redistilled from magnesium turnings. Reactions were stirred magnetically under an argon atmosphere unless otherwise stated.

2,4,6-Trihydroxytoluene (4)
To a solution of 2,4,6-trihydroxybenzaldehyde (**3**) (5.14 g, 33.4 mmol) in THF (80 mL) were added sodium cyanoborohydride (6.29 g, 100.1 mmol) and methyl orange. The mixture was treated with 3M HCl to adjust the pH around 3 and stirred overnight. At completion, the reaction mixture was partitioned between a saturated $NaHCO_3$ solution (100 mL) and ethyl acetate (100 mL). The organic phase was separated, and the aqueous phase was extracted with ethyl acetate (2 × 100 mL). The combined organic layers were washed with brine (50 mL), dried over Na_2SO_4, and concentrated under reduced pressure. The crude was purified by flash chromatography over silica gel (3:1 CH:EE) to give 2,4,6-trihydroxytoluene (**4**) (2.41 g, 52% yield). ^{1}H-NMR (400 MHz, $CDCl_3$): δ (ppm) = 7.83 (s, 2H), 7.72 (s, 1H), 5.97 (s, 2H), 1.99 (s, 3H). ^{13}C-NMR (100 MHz, $CDCl_3$): δ (ppm) = 158.2, 157.5, 103.3, 96.0, 8.7. HPLC-MS (ESI): m/z (%): 139.0 [M^--H] (100), 140.1 [M^-] (16). HR-MS (ESI): *m/z* found = 181.0500, calcd for $C_9H_9O_4$ = 181.0506. IR (cm^{-1}): 3100-3400, 1622, 1538. Rf: 0.58 (EE).

1-(2,4,6-trihydroxy-3-methyl-phenyl)ethenone (5)
To a solution of 2,4,6-trihydroxytoluene (**4**) (2.35 g, 16.8 mmol) in a 10:1 mixture of acetic acid and acetic anhydride (37 mL) was added boron trifluoride etherate (2.13 mL, 17.0 mmol). The resulting mixture was stirred for 3 h at 100 °C. After cooling down the reaction mixture was treated with a 2M NaOH solution to adjust a pH of 4. The reaction mixture was partitioned between water (100 mL) and ethyl acetate (100 mL). The aqueous phase was extracted with ethyl acetate (2 × 100 mL) and the combined organic layer was concentrated. The crude was treated with a 1:1 mixture of 2 M NaOH and MeOH (30 mL) and stirred over night at room temperature. The mixture was neutralized with 3 M HCl and partitioned between water (100 mL) and ethyl acetate (100 mL). The aqueous phase was extracted with ethyl acetate (2 × 100 mL). The combined organic layers were washed with brine (50 mL), dried over Na_2SO_4, and concentrated under reduced pressure. The crude product was purified by flash chromatography over silica gel (2:1 CH:EE) to afford 1-(2,4,6-trihydroxy-3-methyl-phenyl)ethenone (**5**) (974 mg, 32% yield). ^{1}H-NMR (400 MHz, $CDCl_3$): δ (ppm) = 13.77 (s, 1H), 9.53 (s, 1H), 8.99 (s, 1H), 6.08 (s, 1H), 2.62 (s, 3H), 1.97 (s, 3H). ^{13}C-NMR (100 MHz, $CDCl_3$): δ (ppm) = 204.4, 165.6, 163.7, 161.3, 105.9, 104.0, 95.5, 33.4, 8.1. HPLC-MS (ESI): m/z (%): 181.1 [M^--H] (100), 182.1 [M^-] (10). HR-MS (ESI): *m/z* found = 139.0400, calcd for $C_7H_7O_3$ = 139.0401. IR (cm^{-1}): 3000-3500, 2928, 1629, 1607, 1566, 1526. Rf: 0.57 (EE).

1-[2-Hydroxy-4,6-bis(methoxymethoxy)phenyl]ethenone (7)
To a solution of 2,4,6-trihydroxyacetophenone (**6**) (5.32 g, 28.6 mmol) in DCM (100 mL) were added *N*,*N*-diisopropylethylamine (25 mL, 147 mmol) and chloromethyl methyl chloride (4.7 mL, 61.9 mmol). The reaction mixture was stirred overnight. At completion, a saturated NH_4Cl solution (100 mL) was

added and the aqueous phase was extracted with ethyl acetate (3 × 100 mL). The combined organic phases were washed with brine (50 mL), dried over Na_2SO_4, and concentrated under reduced pressure. The crude product was purified by flash chromatography over silica gel (4:1 CH:EE) to give 1-[2-hydroxy-4,6-bis(methoxymethoxy)phenyl]ethenone (**7**) (3.90 g, 53% yield). ^{1}H-NMR (400 MHz, $CDCl_3$): δ (ppm) = 13.72 (s, 1H), 6.28 (d, *J* = 2.3 Hz, 1H,), 6.26 (d, *J* = 2.3 Hz, 1H), 5.27 (s, 2H), 5.19 (s, 2H), 3.54 (s, 3H), 3.49 (s, 3H), 2.67 (s, 3H). ^{13}C-NMR (100 MHz, $CDCl_3$): δ (ppm) = 203.2, 166.8, 163.4, 160.3, 106.9, 97.2, 94.5, 94.0, 56.7, 56.4, 32.9. HPLC-MS (ESI): m/z (%): 255.1 [M^--H] (100), 256.1 [M^-] (14). HR-MS: *m/z* found = 255.0876, calcd for $C_{12}H_{15}O_6$ = 255.0874. IR (cm^{-1}): 3006, 2962, 2909, 2835, 1615, 1590. Rf: 0.71 (CH/EE 1:1).

1-[2,6-Dihydroxy-4-(methoxymethoxy)phenyl]ethenone (8)

Iodine (1.02 g, 4.0 mmol) was added to a solution of 1-[2-hydroxy-4,6-bis(methoxymethoxy)-phenyl]ethenone (**7**) (4.11 g, 16.0 mmol) in methanol (100 mL). The reaction mixture was stirred overnight. After completion, a solution of $Na_2S_2O_3$ (10%) was added till discoloration. The mixture was partitioned between water (100 mL) and ethyl acetate (100 mL). The aqueous phase was extracted with ethyl acetate (2 × 100 mL). The combined organic phases were washed with brine (50 mL), dried over Na_2SO_4, and concentrated under reduced pressure. The crude material was purified by flash chromatography over silica gel (2:1 CH:EE) to obtain 1-[2,6-dihydroxy-4-(methoxymethoxy)-phenyl]ethenone (**8**) (1.96 g, 58% yield). ^{1}H-NMR (400 MHz, Aceton-d_6): δ (ppm) = 11.69 (s, 2H), 6.10 (s, 2H), 5.20 (s, 2H), 3.44 (s, 3H), 2.65 (s, 3H). ^{13}C-NMR (100 MHz, Aceton-d_6): δ (ppm) = 204.9, 165.7, 165.2, 107.2, 96.7, 95.4, 57.1, 33.6. HPLC-MS (ESI): m/z (%): 211.1 [M^--H] (100), 212.1 [M^-] (11.4). HR-MS (ESI): *m/z* found = 211.0611, calcd for $C_{10}H_{11}O_5$ = 211.0612. IR (cm^{-1}): 3234, 3009, 2973, 1634, 1584, 1514. Rf: 0.18 (CH/EE 8:2).

1-[7-Hydroxy-5-(methoxymethoxy)-2,2-dimethyl-chromen-8-yl]ethenone (9)

To a solution of 1-[2,6-dihydroxy-4-(methoxymethoxy)phenyl]ethenone (**8**) (1.94 g, 9.14 mmol) in DCM (100 mL) were added ethylenediamine diacetate (325 mg, 1.83 mmol) and 3-methyl-2-butenal (1.9 mL, 20.1 mmol). The mixture was stirred overnight. At completion, a saturated NH_4Cl solution (20 mL) was added and the mixture was partitioned between water (100 mL) and ethyl acetate (100 mL). The aqueous layer was extracted with ethyl acetate (2 × 100 mL). The combined organic phases were washed with brine (50 mL), dried over Na_2SO_4, and concentrated under reduced pressure. The crude product was purified by flash chromatography over silica gel (4:1 CH:EE) to yield 1-[7-hydroxy-5-(methoxymethoxy)-2,2-dimethyl-chromen-8-yl]ethenone (**9**) (2.19 g, 86% yield). ^{1}H-NMR (400 MHz, $CDCl_3$): δ (ppm) = 13.64 (s, 1H), 6.60 (d, *J* = 10.1 Hz, 1H), 6.21 (s, 1H), 5.45 (d, *J* = 10.1 Hz, 1H), 5.22 (s, 2H), 3.49 (s, 3H), 2.69 (s, 3H), 1.52 (s, 6H). ^{13}C-NMR (100 MHz, $CDCl_3$): δ (ppm) = 203.4, 165.9, 158.4, 156.4, 124.8, 116.6, 106.6, 103.2, 94.9, 94.3, 78.0, 56.5, 33.2, 27.9. HPLC-MS (ESI): m/z (%): 277.1 [M^--H] (100), 278.1 [M^-] (18). HR-MS (ESI): *m/z* found = 278.1077, calcd for $C_{15}H_{17}O_5$ = 278.1081. IR (cm^{-1}): 2963, 2925, 1608, 1584. Rf: 0.59 (CH/EE 8:2).

General procedure for preparation of chalcones 10

To a solution of 1-[7-Hydroxy-5-(methoxymethoxy)-2,2-dimethyl-chromen-8-yl]ethenone (**9**) (1 eq.) in ethanol (15 mL) were added potassium hydroxide (5 eq.) and the aryl aldehyde (1.3 eq.). The reaction mixture was stirred for 3 days at room temperature. At completion, a saturated NH_4Cl-solution was added and the mixture war partitioned between water (50 mL) and ethyl acetate (50 mL). The aqueous layer was extracted with ethyl acetate (2 × 30 mL). The combined organic extracts were washed with brine (15 mL), dried over Na_2SO_4, and concentrated under reduced pressure. The crude products were purified by flash chromatography over silica gel to provide the desired compound (**10**).

(E)-1-[7-Hydroxy-5-(methoxymethoxy)-2,2-dimethyl-chromen-8-yl]-3-phenyl-prop-2-en-1-on (10a)

Compound **10a** was obtained from 1-[7-hydroxy-5-(methoxymethoxy)-2,2-dimethyl-chromen-8-yl]ethenone (**9**) and benzaldehyde. 288 mg, yield: 73%. ^{1}H-NMR (400 MHz, $CDCl_3$): δ (ppm) = 13.98 (s, 1H), 8.13 (d, *J* = 15.6 Hz, 1H), 7.80 (d, *J* = 15.6 Hz, 1H), 7.65-7.62 (m, 2H), 7.47-7.38 (m, 3H), 6.65 (d, *J* = 10.0 Hz, 1H), 6.28 (s, 1H), 5.50 (d, *J* = 10.0 Hz, 1H), 5.25 (s, 2H), 3.52 (s, 3H), 1.58 (s, 6 H). ^{13}C-NMR (100 MHz, $CDCl_3$): δ (ppm) = 193.0, 166.9, 158.7, 155.9, 142.3, 135.6, 130.1, 129.0, 128.3, 127.5, 124.9, 116.8, 107.0, 103.6, 95.3, 94.3, 78.0, 56.5, 28.0. HPLC-MS (ESI): m/z (%): 365.2 [M^--

H] (100), 366.2 [M$^-$] (18). HR-MS (ESI): *m/z* found = 365.1396, calcd for $C_{121}H_{21}O_5$ = 365.1394. IR (cm^{-1}): 3068, 2972, 2958, 2907, 2827, 1642, 1582, 1546. Rf: 0.52 (CH/EE 19:1).

(E)-1-[7-Hydroxy-5-(methoxymethoxy)-2,2-dimethyl-chromen-8-yl]-3-[4-(trifluoromethoxy) phenyl]prop-2-en-1-one (10b)

Product **10b** was obtained from 1-[7-hydroxy-5-(methoxymethoxy)-2,2-dimethyl-chromen-8-yl]ethenone (**9**) and 4-(trifluoromethoxy)benzaldehyde. 293 mg, yield: 89%. ^{1}H-NMR (400 MHz, $CDCl_3$): δ (ppm) = 13.89 (s, 1H), 8.08 (d, *J* = 15.8 Hz, 1H), 7.75 (d, *J* = 15.5 Hz, 1H), 7.64 (d, *J* = 8.7 Hz, 2H), 7.28 (d, *J* = 8.1 Hz, 2H), 6,64 (d, *J* = 9.9 Hz, 1H), 6.28 (s, 1H), 5.51 (d, *J* = 10.0 Hz), 5.25 (s, 2H), 3.52 (s, 3H), 1.57 (s, 6H). ^{13}C-NMR (100 MHz, $CDCl_3$): δ (ppm) = 192.6, 166.9, 158.9, 155.9, 150.2, 140.3, 134.2, 129.5, 128.3, 124.9, 121.2, 120.4 (q, *J* = 258.7 Hz), 116.8, 106.9, 103.6, 95.3, 94.3, 78.1, 56.5, 28.1. HPLC-MS (ESI): m/z (%): 449.2 [M$^-$-H] (100), 450.2 [M$^-$] (20). HR-MS (ESI): *m/z* found = 449.1219, calcd for $C_{23}H_{20}F_3O_6$ = 449.1217. IR (cm^{-1}): 3115, 3079, 2974, 2958, 2915, 2836, 1637, 1587, 1545. Rf: 0.36 (DCM/CH 1:1).

(E)-3-(4-Fluorophenyl)-1-[7-hydroxy-5-(methoxymethoxy)-2,2-dimethyl-chromen-8-yl]prop-2-en-1-one (10c)

Product **10c** was obtained from 1-[7-hydroxy-5-(methoxymethoxy)-2,2-dimethyl-chromen-8-yl]ethenone (**9**) and 4-fluorobenzaldehyde. 194 mg. yield: 62%. ^{1}H-NMR (400 MHz, $CDCl_3$): δ (ppm) = 13.95 (s, 1H), 8.04 (d, *J* = 15.7 Hz, 1H), 7.75 (d, *J* = 15.7 Hz, 1H), 7.59 - 7.63 (m, 2H), 7.10 – 7.16 (m, 2H), 6.34 (d, *J* = 10.0, 1H), 6.28 (s, 1H), 5.50 (d, *J* = 10.0 Hz, 1H), 5.25 (s, 2H), 3.52 (s, 3 H), 1.57 (s, 6H). ^{13}C-NMR (100 MHz, $CDCl_3$): δ (ppm) = 192.8, 166.8, 163.4 (d, *J* = 251.3 Hz), 158.7, 155.8, 141.0, 131.8 (d, *J* = 3.4 Hz), 130.0 (d, *J* = 8.5 Hz), 127.2, 124.8, 116.8, 116.1 116.1 (d, *J* = 21.9 Hz), 106.9, 103.6, 95.3, 94.3, 78.1, 56.5, 28.0. HPLC-MS (ESI): m/z (%): 385.1 [M$^-$-H] (100), 386.1 [M$^-$] (19). HR-MS (ESI): *m/z* found = 407.1264, calcd for $C_{22}H_{21}F_1Na_1O_4$ = 407.1265 IR (cm^{-1}): 2974, 2958, 2906, 1634, 1582, 1544, 1507. Rf: 0.24 (CH/EE 19:1).

(E)-1-[7-Hydroxy-5-(methoxymethoxy)-2,2-dimethyl-chromen-8-yl]-3-(2-thienyl)prop-2-en-1-one (10d)

Product **10d** was obtained from 1-[7-hydroxy-5-(methoxymethoxy)-2,2-dimethyl-chromen-8-yl]ethanon (**9**) and thiophen-2-carbaldehyde. 148 mg, 58% yield. ^{1}H-NMR (400 MHz, $CDCl_3$): δ (ppm) = 14.05 (s, 1H), 8.00 (d, *J* = 15.3 Hz), 7.94 (d, *J* = 7.94 Hz), 7.40 (d, *J* = 4.9 Hz, 1H), 7.32 (d, *J* = 3.44 Hz), 7.10 (dd, *J* = 5.1 Hz, *J* = 3.6 Hz, 1H), 6.63 (d, *J* = 10.4 Hz), 6.26 (s, 1H), 5.50 (d, *J* = 10.4 Hz), 5.24 (s, 2H), 3.51 (s, 3H), 1.61 (s, 6H). ^{13}C-NMR (100 MHz, $CDCl_3$): δ (ppm) = 192.4, 166.9, 158.6, 155.8, 141.4, 135.1, 131.7, 128.3, 128.2, 126.5, 124.5, 116.6, 106.8, 103.6, 95.2, 94.3, 78.1, 56.5, 27.9. HPLC-MS (ESI): m/z (%): 371.2 [M$^-$-H] (100), 372.2 [M$^-$] (17). HR-MS (ESI): *m/z* found = 395.0926, calcd for $C_{20}H_{20}Na_1O_5S_1$ = 395.0924. IR (cm^{-1}): 3326, 3032, 2957, 1709, 1585, 1542. Rf: 0.47 (CH/EE 4:1).

(E)-1-[7-Hydroxy-5-(methoxymethoxy)-2,2-dimethyl-chromen-8-yl]-3-(4-methoxyphenyl)prop-2-en-1-one (10f)

Compound **10e** was obtained from 1-[7-hydroxy-5-(methoxymethoxy)-2,2-dimethyl-chromen-8-yl]ethenone (**9**) and 4-methoxybenzaldehyde. 11 mg, 31% yield. ^{1}H-NMR (400 MHz, $CDCl_3$): δ (ppm) = 14.10 (s, 1H), 8.03 (d, *J* = 15.7 Hz, 1H), 7.79 (d, *J* = 15.7 Hz, 1H), 7.58 (d, *J* = 8.7 Hz, 2H), 6.96 (d, *J* = 8.7 Hz, 2H), 6.64 (d, *J* = 9.8 Hz, 1H), 6.27 (s, 1H), 5.50 (d, *J* = 9.9 Hz, 1H), 5.24 (s, 2H), 3.88 (s, 3H), 3.51 (s, 3H), 1.58 (s, 6H). ^{13}C-NMR (100 MHz, $CDCl_3$): δ (ppm) = 192.9, 166.9, 161.4, 158.5, 155.8, 142.5, 130.0, 128.4, 125.1, 124.8, 116.8, 114.5, 107.0, 103.6, 95.3, 94.3, 77.9, 56.5, 55.4, 28.0. HPLC-MS (ESI): m/z (%): 395.2 [M$^-$-H] (100), 396.2 [M$^-$] (26). HR-MS: *m/z* found = 395.1502, calcd for $C_{23}H_{23}O_6$ = 395.1500. IR (cm^{-1}): 3108, 3065, 2992, 2968, 2937, 1683, 1593, 1546, 1508. Rf: 0.38 (CH/EE 5:1).

General procedure for the deprotection of the MOM-group

Chalcones **10** were dissolved in methanol (30 mL) and a 3 M HCl solution (3 mL) was added. The reaction mixture was refluxed for 2 h. The reaction was quenched with a saturated $NaHCO_3$-solution (15 mL). The reaction mixture was partitioned between water (50 mL) and ethyl acetate (50 mL). The aqueous layer was extracted with ethyl acetate (2 × 30 mL). The combined organic phases were washed

with brine, dried over Na_2SO_4, and concentrated under reduced pressure. The crude products were purified by flash chromatography over silica gel to give the deprotected compounds **11**.

(E)-1-(5,7-Dihydroxy-2,2-dimethyl-chromen-8-yl)-3-phenyl-prop-2-en-1-one (11a)
Compound **11a** was obtained from (E)-1-[7-hydroxy-5-(methoxymethoxy)-2,2-dimethyl-chromen-8-yl]-3-phenyl-prop-2-en-1-one (**10a**). 78 mg, yield: 32%. ^{1}H-NMR (400 MHz, $CDCl_3$): δ (ppm) = 14.11 (s, 1H), 8.12 (d, *J* = 15.6 Hz, 1H), 7.79 (d, *J* = 15.6 Hz, 1H), 7.65-7.60 (m, 2H), 7.47-7.39 (m, 2H), 6.60 (d, *J* = 9.5 Hz, 1H), 6.00 (s, 1H), 5.51 (d, *J* = 9.5 Hz, 1H), 1.58 (s, 6H). ^{13}C-NMR (100 MHz, $CDCl_3$): δ (ppm) = 193.0, 166.5, 158.0, 156.7, 142.4, 135.6, 130.1, 129.0, 128.3, 127.4, 124.9, 116.4, 106.6, 102.4, 96.4, 78.2, 28.0. HPLC-MS (ESI): m/z (%): 321.2 [M^--H] (100), 322.2 [M^-] (20). HR-MS (ESI): *m/z* found = 321.1130, calcd for $C_{20}H_{17}O_4$ = 321.1132. IR (cm^{-1}): 3233, 2973, 1593, 1546. Rf: 0.27 (CH/EE 8:2).

(E)-1-(5,7-Dihydroxy-2,2-dimethyl-chromen-8-yl)-3-[4-(trifluoromethoxy)phenyl]prop-2-en-1-one (11b)
Product **11b** was obtained from (E)-1-[7-hydroxy-5-(methoxymethoxy)-2,2-dimethyl-chromen-8-yl]-3-[4-(trifluoromethoxy)phenyl]prop-2-en-1-one (**10b**). 69 mg, yield: 27%. ^{1}H-NMR (400 MHz, $CDCl_3$): δ (ppm) = 14.02 (s, 1H), 8.08 (d, *J* = 15.7 Hz, 1H), 7.74 (d, *J* = 15.7 Hz), 7.63 (d, *J* = 8.6 Hz, 2H), 7.28 (d, *J* = 8.1 Hz, 2H), 6.60 (d, *J* = 9.9 Hz, 1H), 6.21 (bs, 1H), 6.00 (s, 1H), 5.51 (d, *J* = 9.8 Hz), 1.57 (s, 6H). ^{13}C-NMR (100 MHz, $CDCl_3$): δ (ppm) = 192.6, 166.5, 158.3, 156.7, 150.2, 140.4, 134.2, 129.5, 128.3, 124.8, 121.2, 120.4 (q, *J* = 250.2 Hz), 116.4, 106.6, 102.4, 96.4, 78.3, 28.1. HPLC-MS (ESI): m/z (%): 405.2 [M^--H] (100), 406.2 [M^-] (22). HR-MS (ESI): *m/z* found = 405.0953, calcd for $C_{21}H_{16}F_3O_5$ = 405.0955. IR (cm^{-1}): 3231, 3129, 2971, 2926, 2854, 1628, 1586, 1505. Rf: 0.24 (CH/EE 6:1).

(E)-1-(5,7-Dihydroxy-2,2-dimethyl-chromen-8-yl)-3-(4-fluorophenyl)prop-2-en-1-one (11c)
Product **11c** was obtained from (E)-3-(4-fluorophenyl)-1-[7-hydroxy-5-(methoxymethoxy)-2,2-dimethyl-chromen-8-yl]prop-2-en-1-one (**10c**). 14 mg, yield: 17%. ^{1}H-NMR (400 MHz, $CDCl_3$): δ (ppm) = 14.11 (s, 1H), 8.03 (d, *J* = 15.4, 1H), 7.74 (d, *J* = 15.4, 1H), 7.75 - 7.72 (m, 2H), 7.15 - 7.10 (m, 2H), 6.60 (d, *J* = 9.9 Hz, 1H), 6.01 (s, 1H), 5.50 (d, *J* = 9.9 Hz, 1H), 1.57 (s, 6H). ^{13}C-NMR (100 MHz, $CDCl_3$): δ (ppm) = 192.8, 166.4, 163.8 (d, *J* = 251.3 Hz), 158.4, 156.7, 141.1, 131.8 (d, *J* = 3.6 Hz), 130.0 (d, *J* = 8.5 Hz), 127.2, 124.7, 116.5, 116.1 (d, *J* = 21.9 Hz), 106.5, 102.5, 96.4, 78.2, 28.0. HPLC-MS (ESI): m/z (%): 339.2 [M^--H] (100), 340.2 [M^-] (19). HR-MS (ESI): *m/z* found = 339.1035, calcd for $C_{20}H_{16}F_1O_4$ = 339.1038. IR (cm^{-1}): 3524, 3245, 2970, 2925, 1628, 1595, 1506. Rf: 0.17 (CH:EE 8:2).

(E)-1-(5,7-Dihydroxy-2,2-dimethyl-chromen-8-yl)-3-(2-thienyl)prop-2-en-1-one (11d)
Product **11d** was obtained from (E)-1-[7-hydroxy-5-(methoxymethoxy)-2,2-dimethyl-chromen-8-yl]-3-(2-thienyl)prop-2-en-1-one (**10d**). 43 mg, yield: 36%. ^{1}H-NMR (400 MHz, $CDCl_3$): δ (ppm) = 14.19 (s, 1H), 8.01 (d, *J* = 15.3 Hz, 1H), 7.94 (d, *J* = 15.4 Hz, 1H), 7.40 (d, *J* = 5.3 Hz), 7.32 (d, *J* = 3.5 Hz), 7.10 (dd, *J* = 5.1 Hz, 3.6 Hz, 1H), 6.60 (d, *J* = 9.9 Hz, 1H), 6.43 (bs, 1H), 6.00 (s, 1H), 5.51 (d, *J* = 10.0 Hz, 1H), 1.62 (s, 6H,). ^{13}C-NMR (100 MHz, $CDCl_3$): δ (ppm) = 191.2, 166.6, 158.2, 156.6, 141.5, 135.2, 131.2, 128.3, 128.2, 126.5, 124.9, 116.4, 106.4, 102.4, 96.4, 78.3, 28.0. HPLC-MS (ESI): m/z (%): 327.1 [M^--H] (100), 328.1 [M^-] (18). HR-MS (ESI): *m/z* found = 326.0695, calcd for $C_{18}H_{15}O_4S_1$ = 326.0697. IR (cm^{-1}): 3244, 3105, 3089, 2964, 2922, 1642, 1593, 1547, 1503. Rf: 0.30 (CH/EE 9:1).

General procedure for reacting Eschenmoser’s salt with chromene building blocks 11
To a solution of the deprotected chalcone (1 eq.) in chloroform (15 mL) was added Eschenmoser´s salt (3 eq.). The reaction mixture was stirred for 3 h. The reaction mixture was then diluted with chloroform and a 1M HCl solution (10 mL) was added. The aqueous phase was extracted with ethyl acetate (2 × 25 mL). The combined organic phases were washed with brine (10 mL), dried over Na_2SO_4, and concentrated under reduced pressure. The crude products **12** were used directly without further purification.

[5,7-Dihydroxy-2,2-dimethyl-8-[(E)-3-phenylprop-2-enoyl]chromen-6-yl]methyl-dimethyl-ammoniumiodide (12a)
Compound **12a** was obtained from (E)-1-(5,7-dihydroxy-2,2-dimethyl-chromen-8-yl)-3-phenyl-prop-2-en-1-one (**11a**) and Eschenmoser´s salt. 86 mg, yield: 79%. HPLC-MS (ESI): m/z (%): 380.2 [M^++H-

HI] (34), 381.2 [M^+-HI] (7), 335.1 [M^++H-HI-C_2H_8N] (100). HR-MS (ESI): *m/z* found = 380.1856, calcd for $C_{23}H_{26}N_1O_4$ = 380.1856.

[5,7-Dihydroxy-2,2-dimethyl-8-[(E)-3-[4-(trifluoromethoxy)phenyl]prop-2-enoyl]chromen-6-yl]methyl-dimethyl-ammoniumiodide (12b)

compound **12b** was obtained from (E)-1-(5,7-dihydroxy-2,2-dimethyl-chromen-8-yl)-3-[4-(trifluoromethoxy)phenyl]prop-2-en-1-one (**11b**) and Eschenmoser´s salt. 64 mg, yield: 98%. HPLC-MS (ESI): m/z (%): 462.2 [M^--2H-HI] (100), 463.2[M^--H-HI] (23), 464.2 [M^--HI] (3). HR-MS (ESI): *m/z* found = 464.1677, calcd for $C_{24}H_{25}F_3N_1O_5$ = 464.1679.

[8-[(E)-3-(4-Fluorophenyl)prop-2-enoyl]-5,7-dihydroxy-2,2-dimethyl-chromen-6-yl]methyl-dimethyl-ammoniumiodide (12c)

Compound **12c** was obtained from (E)-1-(5,7-dihydroxy-2,2-dimethyl-chromen-8-yl)-3-(4-fluorophenyl)prop-2-en-1-one (**11c**) and Eschenmoser´s salt. 32 mg, yield 83%. HPLC-MS (ESI): m/z (%): 339.2 [M^--H-HI-C_2H_8N] (100), 340.2 [M^--HI] (19). HR-MS (ESI): *m/z* found = 339.1035, calcd for $C_{20}H_{16}F_1O_4$ = 339.1038.

[5,7-Dihydroxy-2,2-dimethyl-8-[(E)-3-(2-thienyl)prop-2-enoyl]chromen-6-yl]methyl-dimethyl-ammoniumiodide (12d)

Compound **12d** was obtained from (E)-1-(5,7-dihydroxy-2,2-dimethyl-chromen-8-yl)-3-(2-thienyl)prop-2-en-1-one (**11d**) and Eschenmoser´s salt. 36 mg, yield: 53%. HPLC-MS (ESI): m/z (%): 384.2 [M^--H-HI] (100), 385.2 [M^--HI] (26.6). HR-MS (ESI): *m/z* found = 386.1420, calcd for $C_{21}H_{24}N_1O_4S_1$ = 386.1421.

General procedure for couplings via Eschenmoser’s salts 12

A solution of the salt **12** (1 eq.) and 1-(2,4,6-trihydroxy-3-methyl-phenyl)ethenone (1 eq.) in toluene was stirred for 1 h at 110 °C. At completion, the reaction mixture was concentrated under reduced pressure. The crude products were purified by flash chromatography over silica gel to obtain the final compound (**1**, **13**).

(E)-1-[6-[(3-acetyl-2,4,6-trihydroxy-5-methyl-phenyl)methyl]-5,7-dihydroxy-2,2-dimethyl-chromen-8-yl]-3-phenyl-prop-2-en-1-one, rottlerin (1)

Rottlerin (**1**) was obtained from 1-(2,4,6-trihydroxy-3-methyl-phenyl)ethenone (**5**) and [5,7-dihydroxy-2,2-dimethyl-8-[(E)-3-phenylprop-2-enoyl]chromen-6-yl]methyl-dimethyl-ammoniumiodid (**12a**). 25 mg, yield: 29%. ^{1}H-NMR (400 MHz, $CDCl_3$): δ (ppm) = 8.20 (d, 15.8 Hz, 1H), 7.85 (d, *J* = 15.8 Hz, 1H), 7.65-7.60 (m, 2H), 7.47-7.39 (m, 3H), 6.68 (d, *J* = 9.8 Hz, 1H), 5.50 (d, *J* = 9,8 Hz), 3.82 (s, 2H), 2.73 (s, 3H), 2.10 (s, 3H), 1.56 (s, 6H). ^{13}C-NMR (100 MHz, $CDCl_3$): δ (ppm) = 204.1, 192.9, 162.8, 160.6, 159.6, 158.8, 156.6, 155.4, 143.2, 135.5, 130.3, 129.0, 128.4, 126.8, 125.1, 117.2, 106.5, 106.0, 105.3, 104.2, 103.7, 101.9, 78.2, 32.5, 28.0, 15.8, 7.5. HPLC-MS (ESI): m/z (%): 515.3 [M^--H] (100), 516.3 [M^-] (30). HR-MS (ESI): *m/z* found = 515.1713, calcd for $C_{30}H_{28}O_8$ = 515.1711. IR (cm^{-1}): 3234, 2954, 2922, 2851, 1710, 1603, 1555. Rf: 0.24 (CH/EE 2:1).

(E)-1-[6-[(3-Acetyl-2,4,6-trihydroxy-5-methyl-phenyl)methyl]-5,7-dihydroxy-2,2-dimethyl-chromen-8-yl]-3-[4-(trifluoromethoxy)phenyl]prop-2-en-1-one (13b)

Compound **13b** was obtained from [5,7-dihydroxy-2,2-dimethyl-8-[(E)-3-[4-(trifluoromethoxy)phenyl]prop-2-enoyl]chromen-6-yl]methyl-dimethyl-ammoniumiodid (**12b**) and 1-(2,4,6-trihydroxy-3-methyl-phenyl)ethenone (**5**). 6 mg, 25% yield. ^{1}H-NMR (400 MHz, $CDCl_3$): δ (ppm) = 8.16 (d, *J* = 15.5 Hz, 1H), 7.81 (d, *J* = 15.7 Hz, 2H), 7.65 (m, 2H), 7.29 (m, 2H), 6.69 (d, *J* = 9.9 Hz, 1H), 5.51 (d, *J* = 9.9 Hz, 1 H), 3.84 (s, 2H), 2.74 (s, 3H), 2.11 (s, 3H), 1.55 (s, 6H). ^{13}C-NMR (100 MHz, $CDCl_3$): δ (ppm) = 204.1, 192.5, 162.8, 160.6, 159.6, 159.0, 155.4, 150.4, 141.3, 134.1, 129.7, 127.7, 125.1, 121.2, 120.4 (q, *J* = 255.0 Hz) 117.2, 106.5, 105.9, 105.3, 104.2, 103.8, 101.9, 78.2, 32.5, 28.1, 15.8, 7.5. HPLC-MS (ESI): m/z (%): 599.3 [M^--H] (100), 600.3 [M^-] (30). HR-MS (ESI): *m/z* found = 599.1534, calcd for $C_{31}H_{26}F_3O_9$ = 599.134. IR (cm^{-1}): 3246, 2961, 2925, 2855, 1630, 1598, 1507. Rf: 0.10 (CH/EE 2:1).

(E)-1-[6-[(3-Acetyl-2,4,6-trihydroxy-5-methyl-phenyl)methyl]-5,7-dihydroxy-2,2-dimethyl-chromen-8-yl]-3-(4-fluorophenyl)prop-2-en-1-one (13c)

Compound **13c** was obtained from [8-[(E)-3-(4-fluorophenyl)prop-2-enoyl]-5,7-dihydroxy-2,2-dimethyl-chromen-6-yl]methyl-dimethyl-ammoniumiodide (**12c**) and 1-(2,4,6-trihydroxy-3-methyl-phenyl)ethenone (**5**). 7 mg, yield: 21%. ^{1}H-NMR (400 MHz, $CDCl_3$): δ (ppm) = 8.12 (d, *J* = 15.4 Hz, 1H), 7.81 (d, *J* = 15.5 Hz, 1H), 7.64 - 7.59 (m, 2H), 7.16 – 7.11 (m, 2H), 6.79 (d, *J* = 9.9 Hz, 1H), 5.50 (d, *J* = 9.9 Hz, 1H), 3.83 (s, 2H), 2.73 (s, 3H), 2.11 (s, 3H), 1.55 (s, 6H). ^{13}C-NMR (100 MHz, $CDCl_3$): δ (ppm) = 204.1, 192.7, 164.0 (d, *J* = 251.3 Hz), 162.8, 160.6, 159.6, 158.8, 155.4, 142.0, 131.7 (d, *J* = 3.2 Hz), 130.2 (d, *J* = 8.7 Hz), 126.6, 125.0, 117.2, 116.2 (d, *J* = 22.1 Hz), 106.5, 106.0, 105.2, 104.2, 103.8, 101.9, 78.2, 32.5, 28.0, 15.8, 7.4. HPLC-MS (ESI): m/z (%): 533.3 [M^--H] (100), 534.3 [M^-] (30). HR-MS (ESI): *m/z* found = 533,1615 calcd for $C_{30}H_{26}F_1O_8$ = 533.1617. IR (cm^{-1}): 3247, 2954, 2923, 2852, 1599, 1507. 1507. Rf: 0.16 (CH/EE 2:1).

(E)-1-[6-[(3-Acetyl-2,4,6-trihydroxy-5-methyl-phenyl)methyl]-5,7-dihydroxy-2,2-dimethyl-chromen-8-yl]-3-(2-thienyl)prop-2-en-1-one (13d)
Compound **13d** was obtained from [5,7-dihydroxy-2,2-dimethyl-8-[(E)-3-(2-thienyl)prop-2-enoyl]chromen-6-yl]methyl-dimethyl-ammoniumiodide and 1-(2,4,6-trihydroxy-3-methyl-phenyl)ethenone (**5**). 13 mg, yield: 35%. ^{1}H-NMR (400 MHz, $CDCl_3$): δ (ppm) = 8.07 (d, *J* = 15.3 Hz, 1H), 7.98 (d, *J* = 15.3 Hz, 1H), 7.43 (d, *J* = 5.0 Hz, 1H), 7.34 (d, *J* = 3.4 Hz, 1H), 7.10 (dd, *J* = 5.3, 3.4, 1H), 6.67 (d, *J* = 9.7 Hz, 1H), 5.50 (d, *J* = 9.7 Hz, 1H), 3.82 (s, 2H), 2.73 (s, 3H), 2.10, (s, 3H), 1.58 (s, 6H). ^{13}C-NMR (100 MHz, $CDCl_3$): δ (ppm) = 204.1, 192.0, 162.9, 160.6, 159.6, 158.7, 155.3, 141.4, 136.1, 132.0, 128.7, 128.4, 125.8, 125.2, 117.1, 106.5, 106.0, 105.1, 104.3, 103.7, 101.9, 78.3, 32.5, 27.9, 15.8, 7.5. HPLC-MS (ESI): m/z (%): 521.2 [M^--H] (100), 522.2 [M^-] (30). HR-MS (ESI): *m/z* found = 521.1275, calcd for $C_{28}H_{25}O_8S_1$ = 521.1276. IR (cm^{-1}): 3235, 2972, 2927, 1736, 1709, 1600, 1547. Rf: 0.27 (CH/EE 2:1).

Molecular Biology

Materials and Methods

The KCNQ-clones resembles the clone published by NCBI Annotation Project Accession numbers *XM_052604.2* (hKCNQ1) and NM_001127670.3 (hKCNE1). Molecular biological procedures were those described previously (Seebohm G, Sanguinetti MC, Pusch M). In brief. cRNAs were generated by *in vitro* transcription with the Ambion T7 mMessage mMachine kit (Life Technologies. Darmstadt. Germany) from linearized cDNA templates.

Two-electrode voltage-clamp (TEVC) in *Xenopus laevis* oocytes.

The standard TEVC procedures were similar as previously described.[**Error! Bookmark not defined.**] Defolliculated oocytes were obtained from EcoCyte Bioscience (Dortmund. Germany). Oocytes were injected with 8 ng KCNQ1-WT and 4 ng KCNE1-WT and stored for 3-4 days in Bath's solution containing (mmol L-1): 88 NaCl, 1 KCl, 0.4 $CaCl_2$, 0.33 $Ca(NO3)_2$, 0.6 $MgSO_4$, 5 TRIS-HCl, 2.4 $NaHCO_3$ and supplemented with 80 mg L-1 theophylline, 63 mg L-1 benzylpenicillin, 40 mg L-1 streptomycin and 100 mg L-1 gentamycin. Standard TEVC recordings were performed at 22°C with a Turbo Tec 10CX (NPI) amplifier combined with GePulse software for data acquisition. Macroscopic currents were recorded 3-4 days after injection in recording solution ND96 (NaCl 96 mM, KCl 2 mM, $CaCl_2$ 1.8 mM, $MgCl_2$ 1 mM, HEPES 5 mM, pH 7.4). Compound solutions were freshly prepared from 10 mM DMSO stock solutions. For maximal compatibility, all recording solutions including the control solution contained a final DMSO concentration of 0.3 %. Pipettes were filled with 3M KCl and had resistances of 0.5 – 1.5 MΩ.
Channel functions were analysed using the following pulse protocols:

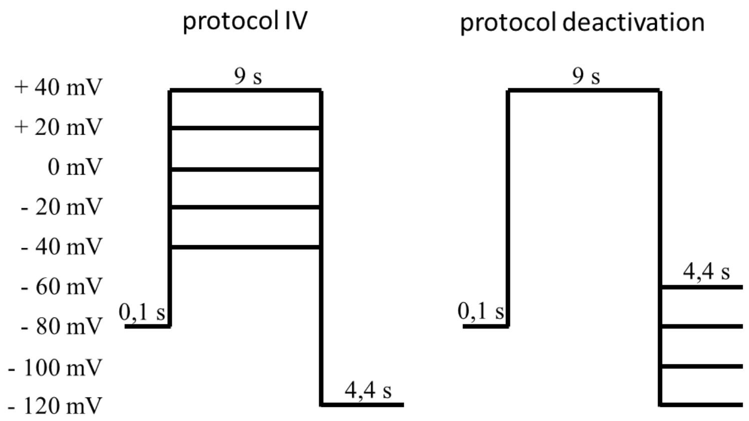

Pulse protocol IV was used to asses the voltage dependent activation of channel currents, while pulse protocol deactivation was used to asses the voltage dependent deactivation of channel currents. Both pulse protocols were sequential applied at the same oocyte to record reference currents (control). After completion, compound solutions were washed in and pulse protocols were applied again under presence of compound solution.

Data analysis

Electrophysiological data were recorded with GePulse and analyzed with accompanying software Ana (Dr. Michael Pusch. Genova. Italy). Data analysis was done using OriginPro 2018 (OriginLab Corporation. Northampton. MA. USA).

Steady-state activation:

Currents were normalized to the average current of the +40 mV pulses in absence of compounds (control) at the end of the 9 sec for recordings of the same experiment (see representative current traces beneath).

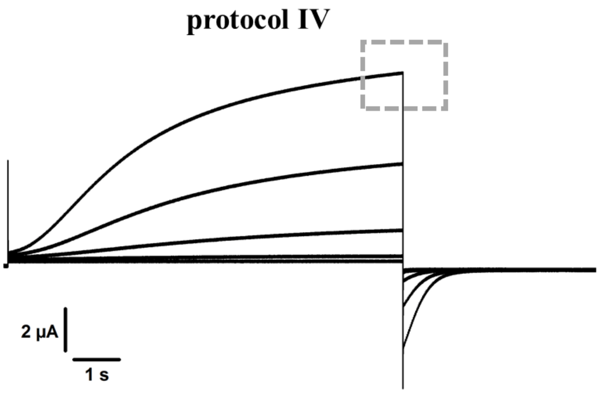

I_{norm} was calculated for every voltage step:

$$I_{norm} = \frac{I_{-\,40\,mV\,-\,+\,40\,mV}}{\overline{I_{+\,40\,mV}}}$$

The resulting normalized currents were fitted to following equitation:

$$Y = ax^2 + bx + c$$

Deactivation:

KCNQ1/KCNE1 channels deactivate due to hyperpolarization using pulse protocol tail current.

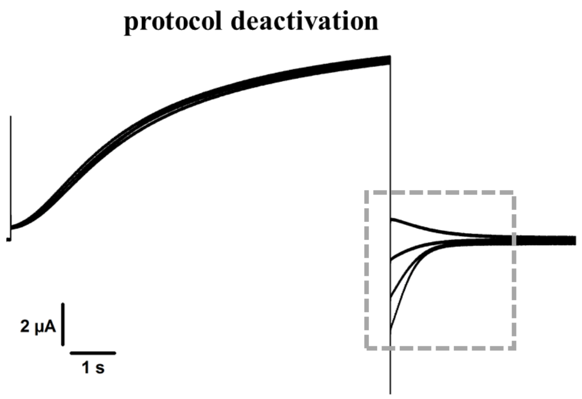

This voltage dependent channel closure can be analyzed by means of exponential fits. For comparison of deactivation kinetics in presence of compounds, deactivation current trace was fitted to following equitation to determine time constant τ:

$$Y = A_0 + A_1 * e^{\frac{-t}{\tau}}$$

Deactivation was fitted for current traces with voltage changes from +40 mV to -120 mV for all oocytes in absence (control) and presence of compounds. After determination of τ_D for all current traces, $\tau_{compound}$ was normalized to $\tau_{control}$:

$$\frac{\tau_{compound}}{\tau_{control}}$$

Statistics:
Statistical significance of results was evaluated by one-way ANOVA with posthoc mean comparison Tukey Test or Student's t test and is indicated by asterisks (ns $p > 0.05$; * $p < 0.05$; ** $p < 0.01$; *** $p < 0.001$).

ACKNOWLEDGEMENTS
The authors thank Ms. I. Polanz, Ms. S. Bettinger and Mr. A. Siebert for measuring the high-resolution MS and NMR spectra.

LITERATURE
